# Sustainable Polyurethane Systems: Integrating Green Synthesis and Closed-Loop Recovery

**DOI:** 10.3390/polym18020246

**Published:** 2026-01-16

**Authors:** Tae Hui Kim, Hyeong Seo Kim, Sang-Ho Lee

**Affiliations:** Department of Chemical and Biochemical Engineering, Dongguk University, Seoul 04620, Republic of Korea

**Keywords:** sustainable polyurethanes, green synthesis, bio-based polyols and isocyanates, chemical recycling, dynamic covalent networks, polyurethane vitrimer, closed-loop materials design

## Abstract

Polyurethanes (PUs) are indispensable polymeric materials widely employed across diverse industrial sectors due to their excellent thermal stability, chemical resistance, adhesion, and mechanical durability. However, the intrinsic three-dimensional crosslinked network that underpins their performance also presents a fundamental barrier to reprocessing and recycling. Consequently, most end-of-life PU waste is currently managed through landfilling or incineration, resulting in significant resource loss and environmental impact. To address these challenges, this review presents an integrated perspective on sustainable PU systems by unifying green synthesis strategies with closed-loop recovery approaches. First, recent advances in bio-based polyols and phosgene-free isocyanate synthesis derived from renewable resources—such as plant oils, carbohydrates, and lignin—are discussed as viable means to reduce dependence on petrochemical feedstocks and mitigate toxicity concerns. Next, emerging chemical recycling methodologies, including acidolysis and aminolysis, are reviewed with a focus on the selective recovery of high-purity monomers. Finally, PU vitrimers and dynamic covalent polymer networks (DCPNs) based on urethane bond exchange reactions are examined as reprocessable architectures that combine thermoplastic-like processability with the mechanical robustness of thermosets. By integrating synthesis, recovery, and reuse within a unified framework, this review aims to outline a coherent pathway toward establishing a sustainable circular economy for PU materials.

## 1. Introduction

Polyurethane (PU) is a representative polymeric material extensively utilized across a broad range of industries, including construction, home appliances, automotive, medical, and textile applications, due to its exceptional durability, flexibility, thermal stability, chemical resistance, and adhesion properties. A key factor enabling PU to achieve both versatility and functionality is the precise tunability of its molecular structure, governed by the combination of polyols and isocyanates [1,2,3,4,5]. Despite these advantages, PU faces a fundamental limitation in recyclability, as it is predominantly a thermosetting polymer that forms three-dimensional crosslinked networks, which render reprocessing and recycling inherently challenging.

Conventional PU synthesis relies on the reaction between polyols and isocyanates in the presence of catalysts, with most precursors derived from fossil resources, thereby contributing to environmental burdens [6,7]. Moreover, the tin-based catalysts commonly used in industrial PU production are subject to increasing regulatory restrictions due to their toxicity, with usage now limited under frameworks established by the International Maritime Organization and the European Union’s R.E.A.C.H. regulation [8]. Simultaneously, most post-consumer PU waste is still disposed of via landfilling or incineration, resulting in environmental pollution, resource depletion, and significant obstacles to realizing a sustainable circular economy. Traditional mechanical recycling methods suffer from performance degradation and downcycling with repeated processing, while thermolysis-based approaches are constrained by high energy demands and the generation of hazardous byproducts. Against this backdrop, the development of sustainable synthesis and recycling strategies that address the full life cycle of PU has emerged as a critical research challenge in polymer and materials chemistry [9].

Most recent review articles on PU sustainability tend to focus on specific stages or isolated strategies within the broader sustainability framework. For example, Rossignolo et al. systematically summarized recent advances in PU recycling technologies; however, their discussion is largely confined to the end-of-life stage, with relatively limited attention to sustainability-driven design at the levels of precursor synthesis or catalyst selection [10]. Similarly, Malucelli and Lorenzetti presented an overview-style review addressing the environmental burden of the PU industry and the necessity of circular economy principles, highlighting the development of naturally derived polyols—such as lignin, vegetable oils, cardanol, and vanillin—as well as bio-based isocyanates, including lysine diisocyanate (LDI) and pentamethylene diisocyanate (PDI). While this work is valuable in outlining macroscopic trends and directions for PU sustainability, the latter sections—covering non-isocyanate PU (NIPU), vitrimers, biodegradable PU, and chemical recycling—remain largely conceptual, with comparatively limited in-depth analysis of reaction mechanisms or process-level implementation strategies [11].

In contrast, the present review offers an integrated perspective that coherently links conventional PU monomers to sustainable synthesis pathways. It begins by systematically outlining the structural characteristics and reaction mechanisms of polyols and isocyanates—the fundamental building blocks of petroleum-based PU—and subsequently discusses transition strategies toward bio-based precursors and NIPU systems. Rather than focusing solely on feedstock substitution, this review emphasizes the chemical diversity of urethane bond formation and the corresponding principles governing property modulation [12].

Furthermore, this review addresses circular recycling strategies with a focus on the post-use stage of PU. In addition to conventional recycling approaches based on depolymerization and resynthesis, it discusses network-preserving strategies that exploit the reversible exchange of urethane bonds. Key reaction mechanisms enabling the recovery of polyols and amines (or isocyanate precursors) via chemical recycling methods—such as acidolysis [13,14], aminolysis [15,16], and glycolysis [17,18]—are summarized, and ongoing research efforts aimed at translating these pathways into practical recycling processes are examined. Dynamic covalent PU networks (PU-CANs) based on transcarbamoylation are also highlighted, with particular attention to differences in network behavior arising from dissociative versus associative exchange mechanisms. Emerging research trends are reviewed in which urethane bond exchange strategies extend beyond stress relaxation and self-healing to enable foam-to-foam closed-loop recycling, thereby preserving both molecular network integrity and porous structures [19,20].

Overall, this review moves beyond the prevailing focus of prior literature on either bio-based material development or isolated recycling technologies by proposing a whole life-cycle integrated framework that links the structural origins of polyurethanes with sustainable synthesis, chemical recycling, and dynamic reprocessing. While earlier studies have primarily outlined the general direction of PU sustainability, the present work consolidates these efforts into a comprehensive review that organizes concrete chemical strategies and actionable technological roadmaps from an operational and system-level perspective. In particular, bio-based polyols, non-isocyanate polyurethanes, dynamic covalent networks, and chemical recycling routes are discussed within a unified framework that emphasizes process feasibility, material circulation, and sustainability-driven design.

## 2. Recent Approaches to PU Synthesis and Sustainable Alternatives

PU is a polymeric material formed through the reaction between polyols and isocyanates. Owing to the high degree of freedom in molecular structure and compositional design, its mechanical, thermal, and chemical properties can be finely tuned across a broad range. These characteristics have enabled PU to become a key material in diverse industrial sectors, including foams, elastomers, coatings, adhesives, and biomedical materials. Consequently, PU remains one of the most important industrial polymers to date [21].

Recent strategies for sustainable PU synthesis can be broadly categorized into two main approaches. The first focuses on improving process safety and environmental compatibility by replacing conventional tin-based catalysts with low-toxicity metal catalysts or organocatalysts, and by developing alternative isocyanate synthesis routes that reduce or eliminate the use of phosgene. The second approach aims to simultaneously enhance feedstock sustainability and reduce toxicity through the incorporation of bio-based polyols and isocyanates derived from renewable resources, or by developing NIPU systems that entirely eliminate the use of isocyanates.

### 2.1. Petroleum-Based PU and Sustainable Alternatives

**Figure 1:**
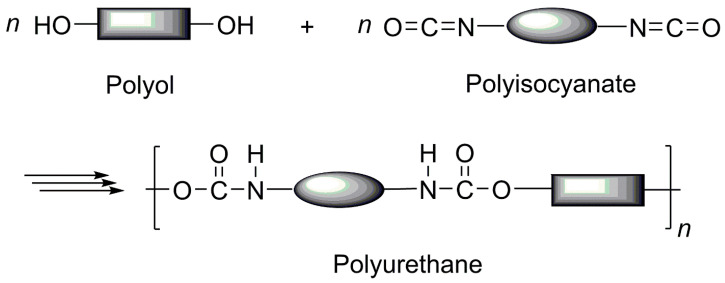
Schematic of PU formation through a polyaddition reaction between polyols and isocyanates. This schematic illustration is intended for conceptual visualization of the urethane-forming reaction and does not represent the relative molecular weights or physical dimensions of polyols and isocyanates.

Petroleum-derived PU systems represent one of the most commercially successful polymer platforms to date, widely utilized across various applications due to their excellent property tunability, processability, and cost competitiveness. PUs are typically synthesized via a polyaddition reaction between polyols and isocyanates (Figure 1) [22]. However, the majority of chemicals used in PU manufacturing are derived from petroleum-based resources, raising increasing concerns about the sustainability and toxicity of PU materials. Accordingly, a thorough understanding of the chemical characteristics of polyols and isocyanates—the core components of petroleum-based PU systems—is essential, along with a critical examination of sustainable alternatives, including phosgene-free isocyanates and replacements for tin-based catalysts.

#### 2.1.1. Structure and Function of Petroleum-Based Polyols

The properties of PU are primarily determined by the chemical characteristics of its constituent components, stoichiometry, and the incorporation of additives. While isocyanates contribute to the chemical tunability of PU, polyols exert the most significant influence on material properties due to their wide structural diversity. As a result, polyols play a central role in enabling a broad range of characteristics, spanning from rigid (granular) to soft (elastomeric) materials and foams. Polyol properties can be tailored by varying the number of terminal hydroxyl (OH), functionality, molecular weight, and backbone architecture (linear or branched). Accordingly, thermoplastic PUs (TPUs) are typically synthesized using polyols with functionalities close to two, whereas thermosetting PU resins—such as foams and structural materials—require polyols with functionalities greater than two.

Another critical parameter influencing PU properties is the molar mass of the polyol. In general, flexible polyols with long chain segments yield soft and elastic polymers, while shorter polyol chains lead to harder materials. This broad property range arises from the intrinsic phase-separated morphology characteristic of PU systems, which typically consist of alternating soft segments derived from polyols and hard segments formed through the reaction of isocyanates with short-chain diols. When low-molecular-weight polyols are used, hydrogen bonding between urethane groups becomes more pronounced, resulting in physical crosslinking that increases rigidity. Therefore, these factors must be carefully considered when designing PU formulations or selecting polyols for specific applications [23,24,25,26,27].

**Figure 2:**
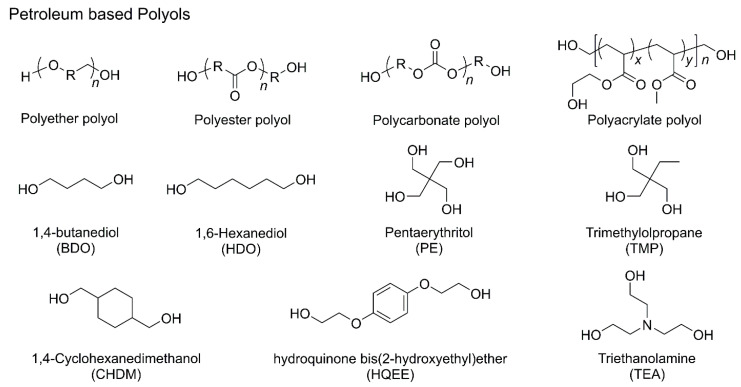
Representative chemical structures of major petroleum-derived polyols commonly used in polyurethane synthesis. The structures shown are illustrative examples selected to highlight key structural classes and functionalities, rather than an exhaustive or quantitative classification.

Most starting materials used in industrial PU production are derived from petrochemical resources (Figure 2) [28]. Petroleum-based polyols are broadly classified into polyether, polyester, polycarbonate, and polyacrylic types.

Polyether polyols, which contain repeating oxygen–carbon bonds, are commonly employed in the synthesis of PU materials for thermoplastic plastics, elastomers, and fibers due to their linear structure (Figure 2). They are typically produced via ring-opening polymerization (ROP) of epoxides such as propylene oxide (PO), ethylene oxide (EO), and butylene oxide. These polyols offer several advantages, including low cost, low viscosity, and the ability to impart hydrolytic stability and flexibility to the resulting PU materials; however, they exhibit relatively poor oxidative and thermal stability [29,30].

Polyester polyol-based PUs display high crystallinity, enhanced thermal stability, and improved flame resistance owing to strong intermolecular interactions between polymer chains. Their primary drawback is their susceptibility to hydrolysis upon prolonged exposure to moisture and heat [31]. Polyester polyols can be synthesized via polycondensation of dicarboxylic acids or through ROP of cyclic carbonates or lactones. Due to the rigidity of ester linkages, PU materials derived from polyester polyols typically exhibit superior mechanical properties compared to those based on polyether polyols; however, their higher viscosity often necessitates synthesis at elevated temperatures [32].

Acrylic polyols represent an important class of polyols widely used in PU production. They are synthesized via radical polymerization under unsaturated conditions to form copolymers, with at least one monomer containing a hydroxyalkyl group to enable reactivity with isocyanates. Their structural diversity allows for versatile property tuning and broad application potential. For example, methyl methacrylate and styrene introduce short-chain structures that impart hardness and water resistance; however, methyl methacrylate is prone to degradation upon light exposure. A notable advantage of methyl methacrylate-containing systems is their ability to become water-soluble after neutralization when functional groups such as carboxylic acids are present. This feature facilitates processing by enabling suitable dispersion and low viscosity in polar solvents or aqueous media [33,34].

Polycarbonate polyols were initially synthesized via the reaction of diols with phosgene; however, a widely adopted route now involves transesterification between glycols and dialkyl or diaryl carbonates at temperatures up to 200 °C. An alternative method is the ROP of cyclic carbonates using appropriate initiators. A representative example is poly(1,6-hexanediol) carbonate, typically obtained through the polycondensation of 1,6-hexanediol and diphenyl carbonate. PUs incorporating polycarbonate polyols exhibit excellent resistance to hydrolysis and oxidation, making them well-suited for long-term applications [35].

Although petroleum-based polyols offer the greatest diversity and tunability, they present inherent limitations from a sustainability standpoint. As a representative alternative, bio-based or renewable-resource-derived polyols have been actively investigated, as discussed in subsequent sections. However, the cost-effectiveness and production reliability of petroleum-based polyols continue to establish them as the industrial standard, and any efforts to replace them must carefully account for economic feasibility.

#### 2.1.2. Structure and Function of Petroleum-Based Isocyanates

**Figure 3:**
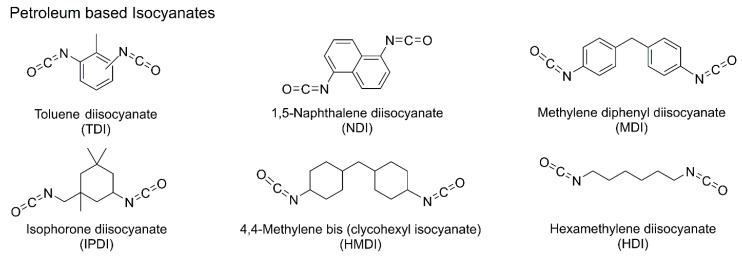
Representative chemical structures of petroleum-derived isocyanates commonly used in PU synthesis.

Isocyanates are compounds containing the –NCO functional group, which reacts with polyols to form urethane linkages. Major petroleum-based isocyanates include methylene diphenyl diisocyanate (MDI), toluene diisocyanate (TDI), hexamethylene diisocyanate (HDI), and isophorone diisocyanate (IPDI) (Figure 3) [31]. MDI, with its aromatic structure, exhibits high reactivity and is suitable for producing rigid materials. TDI, known for its fast reaction rate and excellent compatibility, is primarily used in low-density flexible foams and exists as two isomers: 2,4-diisocyanatotoluene and 2,6-diisocyanatotoluene [36]. In contrast, HDI and IPDI, which are aliphatic and cycloaliphatic isocyanates, respectively, provide enhanced ultraviolet stability, making them particularly suitable for coating applications.

Aromatic isocyanates offer advantages such as rapid reaction kinetics, high ambient-temperature reactivity, good processability, and relatively low cost. They are widely used in rigid PU systems, thermal insulation materials, and TDI-based flexible foams for applications including footwear, cushioning mattresses, and automotive seating. However, aromatic isocyanates are prone to discoloration (yellowing) and exhibit poor weather resistance when exposed to ultraviolet radiation and outdoor environments [31,37,38]. In contrast, aliphatic and cycloaliphatic isocyanates demonstrate superior weather resistance, light stability, and color retention, rendering them more appropriate for coatings and photopolymerization systems. PUs bearing isocyanate end groups tend to be more hydrophobic, chemically stable, and rigid, whereas OH-terminated PUs are generally more hydrophilic, more responsive to external stimuli, and more flexible [21,39].

Isocyanates are typically synthesized via phosgene-based routes, which pose significant safety and toxicity concerns. The –NCO functional group itself is hazardous, presenting irritation and carcinogenic risks upon inhalation or skin contact, thereby necessitating stringent safety management in industrial settings. Commonly used isocyanates, such as TDI and MDI, exhibit high toxicity not only due to their phosgene-based production but also because they can release carcinogenic diamines during degradation. In response to these concerns, phosgene-free synthesis routes and NIPU systems have emerged as promising alternatives.

#### 2.1.3. Non-Phosgene Routes for Isocyanate Synthesis

Most isocyanate precursors are currently produced via phosgene-mediated synthesis, despite the associated risks. Phosgene is an extremely toxic gas with a high potential for leakage and accidental exposure, making worker safety a critical challenge. Additionally, residual phosgene and process-related byproducts, such as HCl and Cl_2_, may be generated during production. Under European Union REACH regulations, the use of phosgene is strictly limited, thereby driving the development of alternative synthetic strategies. In response, several phosgene-free routes have been proposed to eliminate its use and improve the overall safety and environmental profile of isocyanate production.

**Figure 4:**
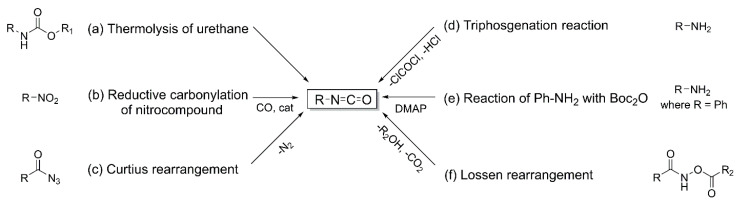
Overview of non-phosgene routes for isocyanate synthesis. (**a**) Thermolysis of urethanes, (**b**) reductive carbonylation of nitro compounds, (**c**) Curtius rearrangement, (**d**) triphosgenation reaction, (**e**) reaction of Ph-NH_2_ with Boc_2_O, and (**f**) Lossen rearrangement.

Thermal decomposition of urethanes requires temperatures above 300 °C under a nitrogen atmosphere, resulting in high energy consumption (Figure 4a) [40]. Additionally, this method is limited by the low volatility of the resulting compounds and poor selectivity. A further drawback is that most starting urethanes used in this approach are themselves derived from phosgene-based isocyanates. However, if urethane bonds are formed through alternative pathways, such as isocyanate–alcohol addition, this strategy may offer a feasible and sustainable route to isocyanates. For instance, the conversion of isophorone diamine (IPDA) to IPDI via urethane pyrolysis has been commercially implemented and patented by Daicel Chemical (Japan); this non-phosgene process affords IPDI in 93% yield, along with approximately 1% isophorone monoisocyanate.

Another approach involves the reductive carbonylation of nitro compounds, which also requires temperatures above 300 °C and pressures exceeding 85 bar, resulting in significant energy consumption (Figure 4b) [41]. This reaction utilizes rhodium or palladium catalysts and produces carbon dioxide as a byproduct. Alternatively, the Curtius rearrangement of carbonyl azides has been investigated to generate isocyanate groups without phosgene (Figure 4c). Proceeding through the thermal decomposition of carbonyl azides, this reaction can be applied to aliphatic, aromatic, and heterocyclic compounds; however, the inherent explosion risk associated with azides severely limits its industrial applicability [42,43,44]. A further method involves the reaction of amines with bis (trichloromethyl carbonate) (Figure 4d). Using triphosgene in a one-step process, functional isocyanate monomers have been synthesized from cardanol. Nevertheless, the generation of phosgene gas during the reaction is unavoidable, and neutralization with aqueous sodium hydroxide is typically required to mitigate residual phosgene toxicity [40]. A particularly attractive route operating under mild conditions is the reaction of aniline derivatives with di-tert-butyl dicarbonate (Boc_2_O) (Figure 4e). This transformation proceeds at room temperature in inert solvents such as dichloromethane, acetonitrile, ethyl acetate, tetrahydrofuran, or toluene, in the presence of 4-dimethylaminopyridine (DMAP). However, this method is not compatible with highly reactive compounds such as alkyl amines, as the generated isocyanates rapidly react with unconverted amines to form urea byproducts [45]. As a final method, the Lossen rearrangement is available (Figure 4f), though its application is limited to the synthesis of aliphatic isocyanates [46].

Although these synthetic routes offer the advantage of producing isocyanates without the direct use of phosgene, phosgene-based processes remain more cost-effective due to their industrial maturity. Moreover, many alternative routes depend on hazardous starting materials, such as triphosgene, azides, or carbon monoxide. As a result, phosgene-based isocyanate synthesis is unlikely to be readily abandoned at the industrial scale. Nevertheless, these alternative strategies have demonstrated effectiveness in reducing the life-cycle toxicity associated with isocyanates [40].

#### 2.1.4. Tin Catalyst Replacement Strategies: New-Generation Catalysts

**Figure 5:**
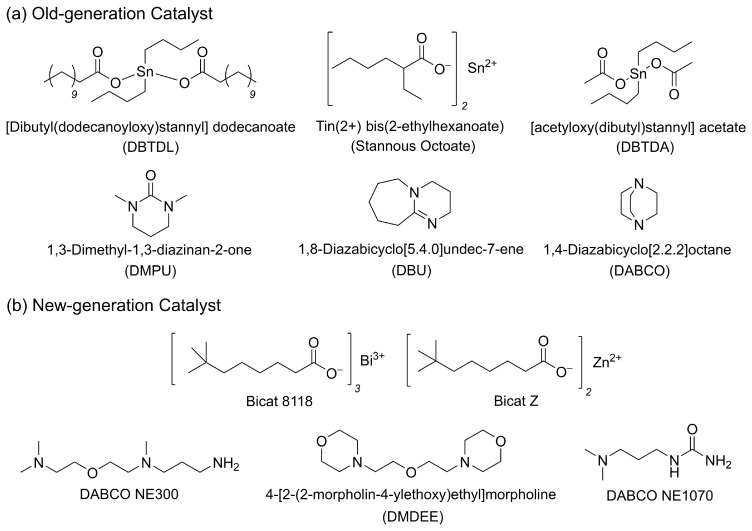
Representative chemical structures of PU catalysts. (**a**) Conventional tin-based and amine catalysts commonly employed in traditional PU formulations. (**b**) Next-generation environmentally benign catalysts, including bismuth- and zinc-based carboxylates, as well as advanced organic amine catalysts.

Catalysts are essential in PU synthesis, as they accelerate reaction rates, improve selectivity, and contribute to energy efficiency. Traditionally, the most widely used catalysts include dibutyl(dodecanoyloxy)stannyl dodecanoate (DBTDL), stannous octoate, 1,4-diazabicyclo [2.2.2]octane (DABCO), and 1,8-diazabicyclo [5.4.0]undec-7-ene (DBU) (Figure 5a). These catalysts exhibit high activity and excellent control over reaction kinetics. However, tin-based catalysts such as DBTDL are associated with significant toxicity concerns and are increasingly being phased out due to regulatory restrictions, including RoHS and REACH. Residual catalysts within the PU matrix can induce adverse effects and promote the formation of toxic compounds, posing a critical drawback, particularly for biomedical applications. Nonetheless, limited use of hazardous catalysts may still be permitted. For instance, morpholine diethyl ether serves as an efficient blowing catalyst in PU foam (PUF) systems, and up to 10% of this organocatalyst can be employed without triggering hazardous pictograms on the material safety data sheet [40].

As a result, there is a growing trend toward replacing toxic tin-based catalysts with safer and more environmentally benign alternatives. Among these, bismuth- and zinc-based carboxylate catalysts are notable for effectively promoting the reaction between polyisocyanates and polyols without inducing harmful side reactions. Bismuth decanoate and zinc decanoate, commercially available under the trade names Bicat 8118 and Bicat Z, respectively, are not classified as substances posing health or physical hazards. Lazzeri et al. introduced a new class of environmentally friendly catalysts that can chemically bind to the PU matrix, thereby reducing catalyst migration and emissions [47]. Representative examples include 3-dimethylaminopropyl urea (trade name: DABCO NE1070, gelling agent) and N-[2-[2-(dimethylamino)ethoxy]ethyl]-N-methyl-1,3-propanediamine (trade name: DABCO NE300, blowing agent) (Figure 5b). These molecules also contain non-reactive amine functionalities that may be released from foams after formation.

The environmentally benign catalysts described above offer viable alternatives to conventional toxic systems for urethane formation. Tin-based catalysts are progressively being replaced by bismuth- and zinc-based systems, while allowable concentrations of previously approved catalysts continue to be reduced. Moreover, catalysts currently classified as non-hazardous may be reclassified as hazardous in the future, prompting stricter usage regulations. Therefore, the development of catalysts that are safe, efficient, and capable of selectively promoting simultaneous polyol–isocyanate reactions remains a critical need [40].

### 2.2. Bio-Based Building Blocks for Sustainable PUs

To develop sustainable PU systems, extensive research has focused on reducing dependence on petroleum-based feedstocks while addressing environmental and health concerns. In this context, bio-based building blocks have emerged as essential components for next-generation PU materials.

A wide range of renewable resources has been explored for the production of bio-based polyols, including vegetable oils, microalgae, lignocellulosic biomass, and polysaccharides. Biomass offers a rich diversity of chemical structures, enabling the synthesis of various polyol types. Lipids have traditionally been investigated as feedstocks for the production of polyester polyols, whereas polysaccharides are more commonly utilized for synthesizing polyether polyols. Lignocellulosic biomass has also been explored as a source of aromatic polyols. Among these, vegetable oil-derived polyols have gained the most attention in both academic and industrial research [27].

Bio-based isocyanates represent another critical class of building blocks for sustainable PU systems, with research primarily focused on securing biomass-derived amine precursors. Structurally diverse renewable resources—such as fatty acids, lignin, amino acids, sugars, and cashew nut shell liquid (CNSL)—offer viable pathways for the synthesis of aliphatic, cycloaliphatic, and aromatic isocyanates. Several of these systems have demonstrated properties comparable to those of conventional petroleum-based isocyanates [1].

However, the synthesis of bio-based isocyanates often involves multistep reaction pathways, resulting in limitations related to process complexity, stability, and yield. Consequently, the industrial implementation of bio-based isocyanates remains at an early stage compared to bio-based polyols, which have reached a relatively mature level of development. Nonetheless, due to their advantages in biocompatibility, resistance to yellowing, reduced toxicity, and lower carbon footprint, bio-based isocyanates are gaining increasing significance, particularly in medical and other high-value PU applications.

#### 2.2.1. Bio-Based Polyols: Aliphatic and Aromatic Structures

Bio-based polyols, primarily derived from vegetable oils, carbohydrates, lignin, and phenolic biomass, are rapidly emerging as sustainable building blocks capable of replacing conventional petroleum-based polyols. These materials are generally classified into aliphatic and aromatic categories based on their molecular structures, each exerting distinct effects on the mechanical, thermal, and optical properties of PUs.

Aliphatic polyols are composed of linear or branched alkyl chains, which impart high chain mobility and low glass transition temperatures (*T*_g_). This structural flexibility enhances softness, elongation at break, energy absorption, and low-temperature elasticity in the resulting PU materials. Additionally, aliphatic polyols offer advantages in transparency and ultraviolet (UV) stability. Aliphatic-based PUs typically exhibit good processability, low viscosity, and improved resistance to yellowing during prolonged use, making them particularly well-suited for coatings and elastomer applications.

In contrast, aromatic polyols contain cyclic π-electron structures, such as phenyl rings, which confer high rigidity and thermal stability. These structural features contribute to elevated *T*_g_, enhanced compressive strength, dimensional stability, heat resistance, and flame retardancy, making them well-suited for rigid foams, high-hardness coatings, and structural applications. However, aromatic structures are susceptible to photo-oxidation under UV exposure, resulting in discoloration and yellowing, while their cyclic nature can lead to increased viscosity and reduced processability [32,48,49,50,51,52].

Thus, aliphatic and aromatic bio-based polyols exhibit complementary structural and property characteristics, offering a versatile platform for the tailored design of PU properties through the strategic selection of diverse biomass-derived feedstocks and appropriate chemical transformation strategies [35].

Polyesters represent one of the most widely utilized classes of bio-based polyols in the PU industry and are structurally defined by R–COO–R′ ester linkages, where R and R′ denote aliphatic or aromatic moieties [53]. To date, polycondensation between diacids and diols remains the most extensively studied and industrially employed method for polyester synthesis. In efforts to transition toward bio-based polyester polyols, numerous renewable diol and diacid monomers have been investigated across both academic and industrial sectors [54]. Linear diols such as ethylene glycol, 1,3-propanediol, 1,4-butanediol, 1,3-butanediol, and 1,4-cyclohexanedimethanol have been explored to increase the bio-based content of polyester polyols [55,56,57,58]. When polymerized with renewable diacids such as succinic acid (SA), adipic acid (AA), or citric acid, these diols enable the synthesis of fully bio-based polyols. Over the past several decades, vegetable oil-based polyols have been extensively studied for the production of bio-based PU materials. These renewable feedstocks accounted for a market size of approximately USD 7.2 billion in 2019 and represent the largest market share among bio-based polyols, with an annual growth rate exceeding 8%.

Vegetable oils are among the most widely utilized feedstocks for bio-based polyol production, as OH functionalities can be introduced through various chemical transformations of the double bonds present in their triglyceride structures [59]. These oils may exhibit relatively uniform structures in the case of homotriglycerides; however, structural variations can occur depending on the botanical source of the oil. With the exception of ricinoleic acid and lesquerolic acid, most fatty acids lack inherent OH groups and thus require functionalization to introduce OH functionalities. This functionalization is typically achieved via chemical modification at the unsaturated sites of the fatty acid chains. Various methods have been investigated for this purpose, including ozonolysis, thiol–ene coupling, hydroformylation, and epoxidation followed by ring-opening reactions, the latter of which is the most widely implemented at the industrial scale. Although all vegetable oil feedstocks are renewable, rapeseed oil, palm oil, and soybean oil are the most abundantly produced and cost-effective, making them particularly suitable for large-scale polyol production [27].

Among various bio-based polyols, castor oil and its derivatives have attracted significant attention due to their intrinsic hydroxyl functionality, renewable origin, and commercial availability. Unlike many other vegetable oils, castor oil contains naturally occurring hydroxyl groups, enabling direct utilization in polyurethane synthesis without extensive chemical modification. As a result, castor-oil-based polyols have been widely applied in flexible and rigid foams, coatings, and elastomers, representing one of the most industrially mature bio-based polyurethane systems [4,27].

**Figure 6:**
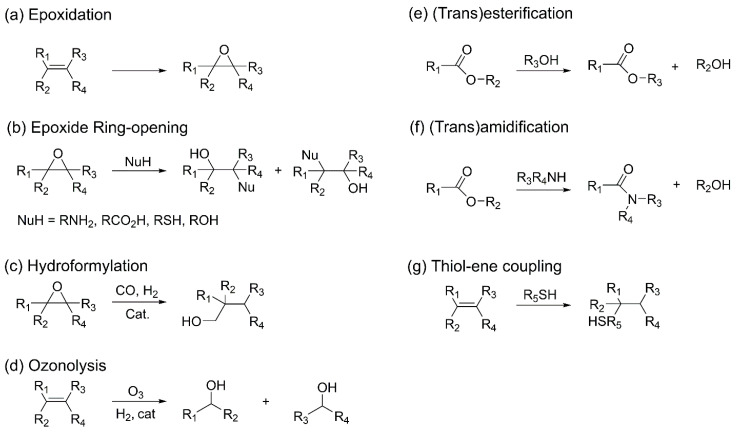
General reaction pathways for the functionalization of vegetable oil-derived feedstocks for the synthesis of bio-based polyols. (**a**) Epoxidation, (**b**) epoxide ring-opening, (**c**) hydroformylation, (**d**) ozonolysis, (**e**) transesterification, (**f**) transamidation, and (**g**) thiol–ene coupling.

Epoxidation followed by epoxide ring-opening is a widely employed strategy for introducing OH groups into bio-based polyols. In this method, peracids are first generated in situ from formic acid, acetic acid, and hydrogen peroxide, and subsequently used to epoxidize carbon–carbon double bonds in the triglyceride structure. The resulting epoxide rings are then opened by nucleophiles such as alcohols, amines, or thiols to introduce OH functionalities (Figure 6a) [60,61,62,63]. Alternatively, once epoxide rings are formed, secondary OH groups can be generated via hydrogenation using hydrogen gas in the presence of a Raney nickel catalyst [64,65,66]. The properties of the resulting polyols are influenced by both the molecular structure of the starting oil and the type of nucleophile employed during ring-opening (Figure 6b). The position and number of OH groups, as well as any residual unsaturation, can significantly affect the mechanical and chemical properties of the resulting PU materials. For example, PUs with high tensile strength and modulus can be synthesized from polyols with a high degree of unsaturation, whereas the use of monoalcohols for ring opening introduces secondary OH groups, leading to reduced reactivity [67,68].

Hydroformylation typically utilizes rhodium- or cobalt-based catalysts to convert carbon–carbon double bonds (C=C) into aldehyde functionalities using synthesis gas (CO/H_2_), followed by hydrogenation of the resulting carbonyl compounds to yield primary OH groups (Figure 6c) [69]. Hydroformylated bio-oils feature highly reactive primary OH groups along the backbone, which significantly enhance curing rates. Additionally, the ability to control chain length and branching during this process allows for a tunable rigidity–flexibility balance. Indeed, vegetable oil-based hydroformylated polyols have been reported to exhibit increased compressive strength and accelerated curing behavior in rigid foam applications.

Ozonolysis involves the reaction of ozone with C=C bonds to generate two aldehyde functionalities, accompanied by the decomposition of ozonides into aldehydes and carboxylic acids (Figure 6d). Subsequent reduction of aldehyde groups to alcohols using a Raney Ni catalyst yields di- or trifunctional polyols. Although ozonolysis of soybean oil, rapeseed oil, and synthetic triglycerides can produce polyols containing highly reactive primary alcohols, this technique is generally limited to the production of polyols with fewer than three functional groups, which may present a drawback for thermoset PU synthesis [70].

Vegetable oils also contain abundant ester functionalities, which can be exploited for polyol synthesis via transesterification reactions with alcohols under organic or inorganic base catalysis. Common alcohols used in this process include pentaerythritol, trimethylolpropane, and glycerol [71,72,73]. Analogously, transamidation can be performed using appropriate amines (Figure 6e,f) [74].

Thiol–ene coupling involves the reaction of thiol alcohols with C=C bonds to form C–S linkages. This process proceeds via a photoinitiated free-radical chain mechanism, wherein fatty acid double bonds react with thiols to generate primary OH groups (Figure 6g). This strategy requires no metal catalysts, imposes minimal constraints on feedstock structure, and operates under mild conditions with high conversion efficiency, making it a widely adopted approach for functionalizing bio-based oils [75,76].

The most common route for polyether synthesis involves the ROP of cyclic ethers, with poly(tetrahydrofuran) (poly(THF)) representing one of the most widely produced examples. BASF has proposed a bio-based poly(THF) with properties equivalent to its petrochemical counterpart, synthesized from bio-based 1,4-butanediol produced under license from Genomatica. Another bio-based cyclic ether, isosorbide, is a low-cost monomer derived from sorbitol and is considered one of the most promising bio-based building blocks [27]. Its rigid molecular structure imparts a high *T*_g_, excellent transparency, and strong resistance to ultraviolet radiation, heat, and impact [77,78].

Polysaccharides such as starch, chitosan, and alginate have also been investigated as bio-based polyols for sustainable PU production due to their biocompatibility, biodegradability, and renewability [79,80,81]. These naturally occurring polymers consist of monosaccharide units linked by glycosidic bonds and exhibit structural similarity while offering diverse functional groups, including amines, carboxylic acids, and sulfates [82]. In addition to increasing the bio-based content in PU formulations, polysaccharides have been employed to promote the degradation of the final polymer materials. Among these, starch is the most extensively studied polysaccharide polyol, and crosslinked PUs can be synthesized via reactions between starch and oligomeric diisocyanates [81,83,84]. Chitosan, another notable polysaccharide, is obtained through controlled deacetylation of chitin and contains amine functionalities, which confer inherent antibacterial and antifungal properties—attributes particularly valuable in biomedical applications [85,86].

The self-condensation of bio-sourced diols has also been explored as a route for polyether polyol production. Traditionally, polyether synthesis has relied on Williamson etherification, which involves nucleophilic substitution between alkoxides and alkyl halides [87]. A key advantage of self-condensation, however, is that it generates water as a byproduct, in contrast to Williamson etherification, which produces chloride salts. As a result, there is growing interest in self-condensation approaches for more sustainable polyether synthesis, and several companies have begun commercializing polyethers derived from renewable feedstocks. For example, poly(trimethylene ether glycol) (PO3G) is produced by SK Chemicals from 100% bio-based 1,3-propanediol derived from corn sugar. Similarly, the WeylChem Group has commercialized related products under the trade name Velvetol.

Aromatic polyols are derived from natural aromatic resources such as lignin, cardanol, and vanillin. Incorporating aromatic components into macromolecular structures enhances the mechanical strength, thermal stability, and flame resistance of PU materials [88,89,90].

**Figure 7:**
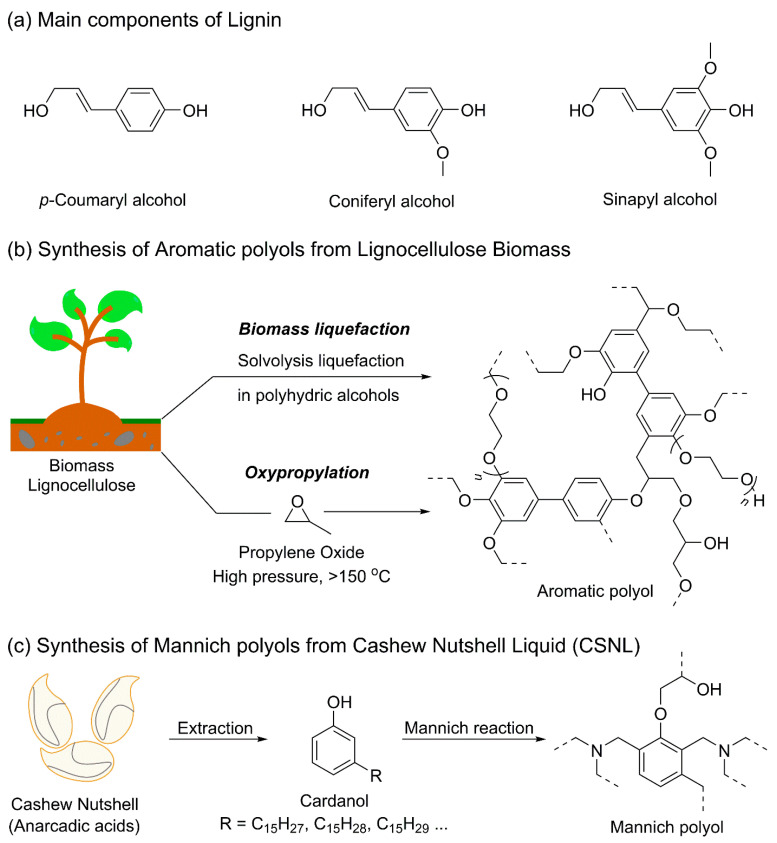
Representative structures and synthesis pathways of aromatic bio-based polyols. (**a**) Major structural units of lignin, (**b**) synthesis of aromatic polyols from lignocellulosic biomass through solvolytic liquefaction followed by oxypropylation, and (**c**) preparation of Mannich-type polyols from CNSL.

Lignin, a naturally occurring polyphenolic substance, constitutes approximately 5–45% of lignocellulosic biomass in wood and annual crops. It is primarily composed of three monolignols—sinapyl alcohol, coniferyl alcohol, and p-coumaryl alcohol—in varying proportions (Figure 7a) [91]. The structural characteristics of lignin depend not only on the botanical source but also on the extraction and fractionation methods employed. Common lignin types include Kraft lignin, lignosulfonate lignin (which contains sulfur), soda lignin, and organosolv lignin.

Effective reactions between phenolic OH groups and isocyanate functionalities are challenging due to steric hindrance, low reactivity, and the pronounced chemical heterogeneity of lignin. Therefore, chemical modification is required to convert lignin into liquid polyols suitable for PU production, a process commonly referred to as liquefaction [92]. Liquefaction enhances the accessibility and reactivity of lignin OH groups through functionalization.

Solvolytic liquefaction of lignocellulosic biomass in the presence of polyhydric alcohols produces aromatic polyols applicable to PU synthesis (Figure 7b). This process cleaves *β*-1,4-glycosidic bonds, thereby separating cellulose from the lignin fraction [93,94]. Liquefaction solvents may include low-molar-mass alcohols such as ethylene glycol, glycerol, and methanol, as well as high-molar-mass polyols such as poly(propylene glycol) and poly(ethylene glycol). The solvent is typically used in excess, and the final OH value of the liquefied polyols depends on the molar mass of the polyhydric solvent, generally ranging from 230 to 1300 mg KOH·g^−1^ [95,96]. Due to their high functionality, these polyols are primarily employed as crosslinking agents in structural materials and foams. In addition to direct liquefaction, alkoxylation is another strategy for producing aromatic polyols from lignin. The most commonly used alkoxylation agent is PO gas [97]. Oxypropylation typically occurs in a closed reactor at 150–180 °C under pressure, wherein lignin reacts with PO [98].

Mannich polyols constitute a class of amine-containing polyether polyols with aromatic structures. They are synthesized via the formation of Mannich bases, followed by subsequent polyol formation. The presence of tertiary amine groups imparts autocatalytic reactivity, enabling sufficient reactivity toward isocyanates without the need for additional catalysts [99]. Bio-based Mannich polyols can be obtained through chemical modification of cardanol, which is derived from aromatic limonene derivatives or CNSL, an inexpensive and renewable resource. The main components of CNSL include anacardic acid (60–65%), cardol (15–20%), cardanol (10%), and methyl cardol [100].

When anacardic acid present in CNSL is converted into the phenolic lipid cardanol, aromatic polyols with high OH values—typically exceeding 200 mg KOH·g^−1^—can be synthesized (Figure 7c) [68,100,101]. Numerous studies have demonstrated the use of cardanol as a renewable feedstock for diol synthesis. Notably, Cardolite, a U.S.-based manufacturer specializing in CNSL-derived products, has developed industrial-scale bio-based Mannich polyols derived from CNSL. Marketed under the trade name GX, Cardolite offers six different Mannich polyols (9101–9106) with average functionalities ranging from 3 to 4. These products are liquid in form, with colors ranging from red to brown, and exhibit viscosities between 1550 and 13,000 cPs at 25 °C [68,99,100]. Mannich polyols are primarily used in the synthesis of rigid PU spray foams with high *T*_g_, typically ranging from 50 to 110 °C. The presence of aromatic rings and high functionality promotes the formation of rigid materials, while their high reactivity and excellent flame and thermal resistance make them particularly suitable for PUF applications. However, some Mannich polyols exhibit high viscosities, which can limit their incorporation into pumped formulations [102].

Given the wide range of available biomass resources—including vegetable oils, lipids, and polysaccharides—numerous polyols with diverse molar masses and functionalities, such as polyethers, polyesters, and polyphenols, have been reported. Although the use of biomass-derived feedstocks for polyol production in the PU industry has been explored in academic literature for several decades, industrial adoption of bio-based polyols has only recently gained momentum. These polyols represent one of the most promising strategies for replacing petroleum-based counterparts. However, the selection between aliphatic and aromatic structures, along with the specific chemical conversion strategies employed, significantly influences the reactivity, processability, material properties, and long-term stability of the resulting PUs [27].

It should be noted that a bio-based origin does not automatically guarantee overall sustainability. Depending on feedstock cultivation, land use change, processing intensity, and transportation, the environmental footprint of bio-based polyols may in some cases be comparable to or even higher than that of petrochemical counterparts. Therefore, sustainability claims should be evaluated from a comprehensive life-cycle perspective.

In addition to environmental considerations, the industrial sustainability of bio-based polyurethanes is strongly influenced by factors such as production cost, reaction yield, solvent consumption, and feedstock variability. In particular, the heterogeneous chemical composition of certain biomass resources, such as lignin, can lead to batch-to-batch variability, posing challenges for process reproducibility and large-scale implementation.

#### 2.2.2. Bio-Based Isocyanates

Bio-based isocyanates constitute a key class of building blocks in the development of sustainable PU systems. Recent research has focused on establishing environmentally benign synthetic routes that avoid phosgene, while also expanding the variety of biomass-derived amine precursors. Structurally diverse biomass sources, including fatty acids, lignin, amino acids, sugars, algae, and CNSL, have been investigated as alternative feedstocks for producing aliphatic, cycloaliphatic, and aromatic isocyanates. Based on these resources, significant progress has been made toward the development of bio-based diisocyanates with performance characteristics comparable to conventional petroleum-derived isocyanates such as HDI, IPDI, and MDI [1].

**Figure 8:**
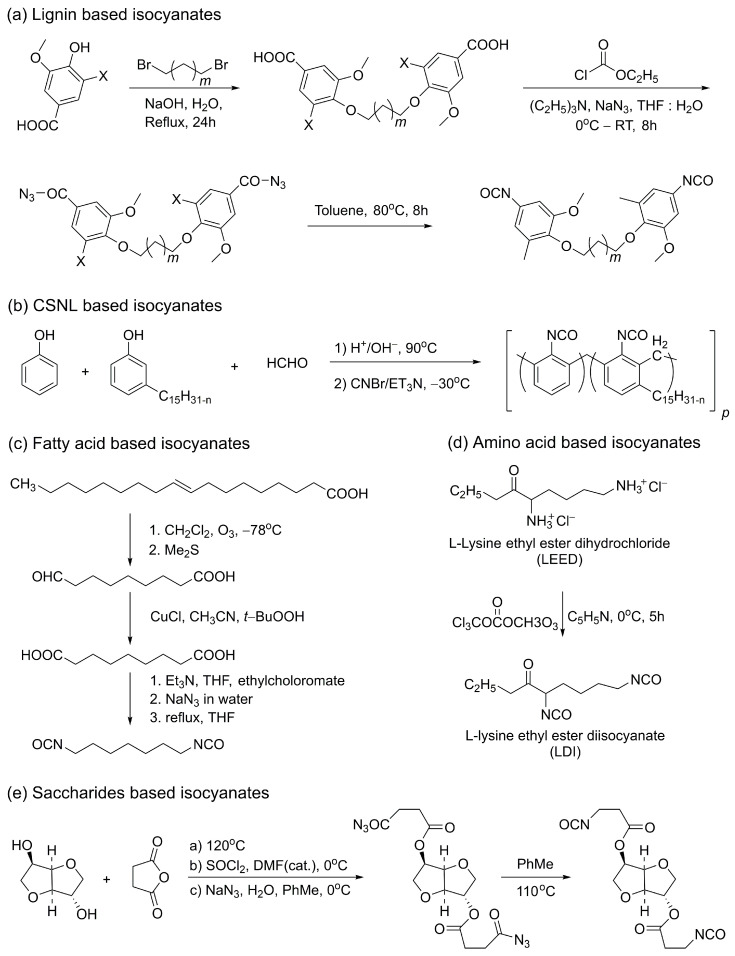
Synthetic routes to bio-based isocyanates derived from various renewable feedstocks. (**a**) lignin-based aromatic diisocyanates synthesized from lignin-derived phenolic acids via Curtius rearrangement, (**b**) CNSL-based isocyanates from cardanol-derived phenolic structures, (**c**) fatty acid-based aliphatic diisocyanates obtained from vegetable oil derivatives, (**d**) amino acid-based diisocyanates exemplified by lysine-derived compounds, and (**e**) saccharide-derived diisocyanates synthesized from sugar-based platform molecules.

Lignin is an important renewable resource due to its abundance and widespread availability [103]. It can be extracted from lignocellulosic materials through various processing methods, with both the extraction source and processing conditions significantly influencing its chemical structure. Additionally, lignin is generated as a byproduct in several industries, such as the cellulose industry, further enhancing its potential for valorization [104]. In recent years, increasing attention has been directed toward the use of lignin-derived aromatic components, including vanillin, syringic acid, vanillic acid, guaiacol, and syringol, in PU synthesis. As discussed previously, lignin contains a high density of OH groups and is therefore frequently utilized as a substrate for renewable polyols. However, lignin has also been investigated as a precursor for isocyanate synthesis. For example, Kuhire et al. reported the synthesis of aromatic diisocyanates, specifically bis(4-isocyanato-2-methoxyphenoxy)alkane and bis(4-isocyanato-2,6-dimethoxyphenoxy)alkane, via Curtius rearrangement (Figure 8a), using lignin-derived vanillic acid and syringic acid as starting materials. These diisocyanates were subsequently employed in the synthesis of poly(ether)urethanes using aliphatic bio-based diols such as 1,10-decanediol and 1,12-dodecanediol [105]. Due to its favorable properties, including solubility, low cost, and biodegradability, lignin has garnered significant interest for use in polymer matrices and other industrial applications [106]. However, the structure and functionality of lignin vary with its source and processing conditions, which can influence the properties of the final products. This inherent variability often results in structural inhomogeneity, posing challenges for commercial applications that require high purity and reproducible performance [107].

CNSL, a byproduct of cashew nut kernel removal, represents an attractive renewable resource. Belonging to the class of natural phenols, CNSL primarily comprises anacardic acid, cardanol, cardol, and 2-methylcardol. Cardanol finds utility in the chemical industry in applications such as coatings, resins, and surfactants [108]. Notably, novolac resins containing cardanol can be converted into the corresponding cyanate esters. Nair et al. investigated the synthesis of cardanol-modified novolac resins and examined the effect of cardanol content on thermal stability (Figure 8b) [109]. The resulting cyanate esters formed thermally stable phenol–triazine networks upon curing; however, increasing the cardanol content reduced the thermal stability of the cured polymers. Kulkarni et al. synthesized cyanate ester monomers containing pentadecyl-substituted cyclohexyl moieties, including 1,1-bis(4-cyanatophenyl)-3-pentadecylcyclohexane and 1,1-bis(4-cyanatophenyl)cyclohexane [110]. These compounds exhibited improved processability, lower melting points, and slower curing rates compared to commercially available cyanate esters.

Fatty acids can also serve as precursors for isocyanate synthesis. Isocyanates derived from vegetable oils typically possess aliphatic structures and exhibit lower reactivity than the aromatic isocyanates obtained from petrochemical feedstocks [111]. Hojabri et al. proposed a route for synthesizing aliphatic 1,7-heptamethylene diisocyanate (HPMDI) from oleic acid via Curtius rearrangement (Figure 8c) [112]. This method involves the thermal decomposition of acyl azides, which act as intermediates in isocyanate production. The resulting diisocyanate displayed physical properties comparable to those of petrochemical-based counterparts. However, a significant drawback of this process is the requirement for strictly anhydrous reaction conditions. Additionally, the azides employed are highly explosive and must be handled at low temperatures, posing challenges for large-scale implementation.

In another study, Hojabri et al. synthesized 1,16-diisocyanatohexadec-8-ene (HDEDI) from oleic acid via Curtius rearrangement [113]. Initially, oleic acid was reacted with a Grubbs catalyst to produce an unsaturated dicarboxylic acid, which was subsequently subjected to Curtius rearrangement using ethyl chloroformate, triethylamine, and dehydrated tetrahydrofuran to yield HDEDI. PUs synthesized from HDEDI exhibited stronger hydrogen bonding than those derived from HPNDI and demonstrated higher tensile strength at break compared to TPUs based on HPMDI. Additionally, HDEDI-based PUs displayed lower Young’s modulus and greater elongation at break. However, the need for large amounts of solvent and extended processing times to separate diols from the polyol mixture hinders the practicality of large-scale production.

Most amino acid-based PUs have been developed for biomedical applications. Jian Han et al. proposed a method for synthesizing bio-based PUs using L-lysine diisocyanate ethyl ester (Figure 8d) [114]. Polymerization was carried out in two steps using 1,4-butanediol and polycaprolactone diols (PCL) [115]. The resulting PUs exhibited a maximum tensile strength of 23 MPa and an elongation at break of up to 1700%. As PCL is a biodegradable polyester, it significantly enhanced the biodegradability of the resulting PUs. The degradation products included non-toxic L-lysine amino acids and α-hydroxycaproic acid; however, the overall yield was limited to approximately 50%. Furthermore, toxic substances such as pyridine, used during synthesis, must be removed prior to industrial application. In a separate study, biodegradable PUs were prepared using a poly(lactic acid)–poly(ethylene glycol)–poly(lactic acid) (PLA–PEG–PLA) triblock oligomer, L-lysine ethyl ester diisocyanate (LDI), and 1,4-butanediol (BDO) in equivalent stoichiometric ratios [116]. The resulting PUs exhibited rapid degradation behavior and showed potential for applications in drug delivery systems and as contrast agents for magnetic resonance imaging.

Sugar biomass extracted from corn, soybean, wheat, and related sources provides an abundant supply of natural sugars suitable for the synthesis of saccharide-derived isocyanates. This biomass offers several advantages, including structural diversity, non-toxicity to humans, hydrophilicity, and environmental compatibility. Polymer production substrates can be derived from saccharides such as mannose, lactose, and galactose [117,118]. Zenner et al. reported the synthesis of a diisocyanate derived from isosorbide (Figure 8e) [119]. Due to its inherent rigidity and thermal stability, isosorbide represents a promising alternative to petroleum-based feedstocks. In their synthesis, succinic anhydride was also employed, as SA can be produced at competitive cost and allows for the elimination of petrochemical reagents. In the first step, isosorbide and succinic anhydride underwent double esterification under solvent-free conditions. The target diisocyanate was then obtained via Curtius rearrangement, using thionyl chloride and sodium azide. A limitation of this approach is the use of aromatic solvents such as toluene during synthesis. The overall yield of the isosorbide-derived diisocyanate was approximately 60%, and the resulting product was successfully applied in the synthesis of thermoplastic PUs.

Bio-based isocyanates can be classified as aliphatic, cycloaliphatic, or aromatic, depending on the feedstock, with each category imparting distinct properties such as flexibility, yellowing resistance, flame retardancy, and biocompatibility. Continued advances in phosgene-free synthesis methods, the availability of biomass-derived diamine precursors, and improvements in biodegradability and carbon footprint reduction are collectively enhancing the commercial viability of fully bio-based PU systems.

### 2.3. NIPUs

As discussed in Section 2.1.2, isocyanates are industrially derived from phosgene-based feedstocks and are associated with significant health risks, including dermatitis and asthma. In response to growing environmental concerns and the increasing adoption of green chemistry principles, NIPUs have recently emerged as viable alternatives to conventional PU systems.

#### 2.3.1. Synthetic Routes for NIPUs

**Figure 9:**
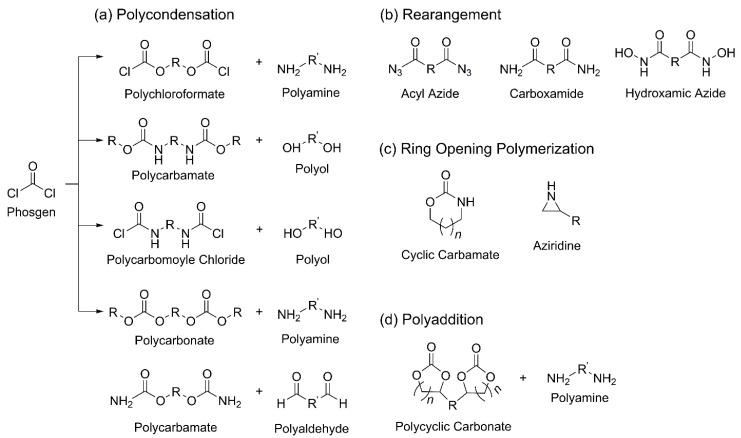
Representative synthetic routes for NIPUs. (**a**) Polycondensation, (**b**) rearrangement, (**c**) ROP, and (**d**) polyaddition.

As illustrated in Figure 9, the synthetic routes for NIPUs can be broadly categorized into four main types: polyaddition, polycondensation, rearrangement, and ROP.

Among the polycondensation-based synthetic routes for NIPUs are reactions between polychloroformates and polyamines, transurethanization between polycarbamates and polyols, reactions of polycarbamoyl chlorides with polyols, reactions between polycarbonates and polyamines, and processes involving polycarbamates (Figure 9a). However, these routes typically require phosgene or its derivatives for precursor synthesis. Moreover, polycondensation reactions often generate byproducts such as alcohols or HCl, which impose limitations on their industrial application [120]. An alternative polycondensation route involves the reaction between polycarbamates and polyaldehydes.

This approach proceeds via reactions among primary carbamates, aldehydes, and acidic catalysts, resulting in the formation of two urethane groups attached to the same carbon atom. The carbamate–aldehyde reaction is considered a promising route for NIPU synthesis, as it avoids the generation of toxic volatile organic compounds (VOCs), offers access to a broad range of commercially available bio-based aldehydes, and enables the use of bio-based polycarbamates derived from bio-based polyols and methyl carbamate. However, further studies are needed to optimize the reaction conditions to reduce reaction time and temperature while achieving higher yields [40].

NIPUs can also be synthesized via rearrangement reactions, including those involving acyl azides (Curtius rearrangement), carboxamides (Hofmann rearrangement), and hydroxamic azides (Lossen rearrangement) (Figure 9b) [35,121]. In these routes, isocyanates are generated in situ and subsequently react with alcohols to form PUs. Although these methods avoid direct handling of isocyanates, a critical limitation is the high hazard level of the precursors involved. Mallia et al. demonstrated the Curtius rearrangement under continuous flow conditions at the kilogram scale, marking progress toward scalability [122]. However, due to the explosion risks associated with azide precursors and the necessity for stringent control over high-temperature conditions involving N_2_/CO_2_ evolution, this approach remains restricted to pilot-scale demonstrations.

Another synthetic route involves the ROP of aliphatic cyclic carbamates or aziridines (Figure 9c) [123,124,125]. However, ROP-based NIPUs have not been widely adopted in large-scale or structural applications and remain limited to specific uses such as adhesives and foams. Although this method offers the advantages of byproduct-free synthesis and simplified purification, it typically requires high reaction temperatures. Additionally, cyclic carbamates are generally synthesized from phosgene, and the aziridines used as monomers pose acute toxicity concerns [126]. To achieve sufficient molecular weight, this polymerization generally requires temperatures above 140 °C. While some recently developed catalysts have reduced the necessary temperature to approximately 110 °C, these milder conditions often result in slower reaction rates or broader molecular weight distributions [127].

Due to the stability and performance limitations of the aforementioned routes, significant research has shifted toward the polyaddition of cyclic carbonates with amines (Figure 9d). This approach avoids the use of toxic isocyanates and phosgene-based precursors, making the process inherently safer, more environmentally sustainable, and suitable for a wider range of applications. For example, Cornille et al. first reported NIPU foams (NIPUFs) synthesized via the reaction of cyclic carbonates with diamines in 2015. The resulting high-density, flexible NIPUFs (194–295 kg m^−3^) demonstrated excellent thermal stability and recoverable compressive behavior. Moreover, cyclic carbonates are non-toxic and, unlike isocyanates, are not moisture-sensitive, thereby preventing the formation of byproducts such as urea or CO. As a result, they do not require special handling or storage precautions [128].

In the polyaddition process, amines act as nucleophiles that attack the carbonyl carbon of cyclic carbonates (five- or six-membered rings), followed by a proton transfer mechanism that generates primary or secondary OH groups. Because OH groups are incorporated along the main polymer backbone, the final products of this route are referred to as poly(hydroxyurethane)s (PHUs), which differ from conventional PUs. Moreover, the presence of OH groups facilitates hydrogen bonding, thereby enhancing chemical resistance to nonpolar solvents. One of the major advantages of PHUs lies in their flexibility in feedstock selection, as a wide variety of cyclic carbonates and amines—derived from biodegradable, renewable, and sustainable resources—can be employed as bio-based precursors [129].

Among the various synthetic pathways for NIPUs, the polyaddition of cyclic carbonates with amines appears to be the most promising. However, PHUs are limited by the low reactivity between cyclic carbonates and amines, restricted reaction progress at ambient temperature, and the resulting low molecular weights. Additional challenges include high viscosity, slow ambient curing, and compliance with stringent VOC limitations. To address these issues, researchers have developed new formulation strategies, including waterborne NIPUs and hybrid NIPU systems [120,128].

#### 2.3.2. Advanced NIPU Systems

Although cyclic carbonate–amine-based polyaddition currently represents the dominant synthetic route in NIPU research, this approach remains constrained by the aforementioned technical limitations, particularly reduced reactivity and low molecular weight. To overcome these challenges and meet the performance requirements of diverse industrial applications, several advanced NIPU strategies have been developed. Among them, hybrid NIPUs, waterborne NIPUs (WNIPUs), and NIPUFs have emerged as the most actively investigated, representing key approaches to compensate for the structural shortcomings of conventional PHU-based NIPUs.

**Figure 10:**
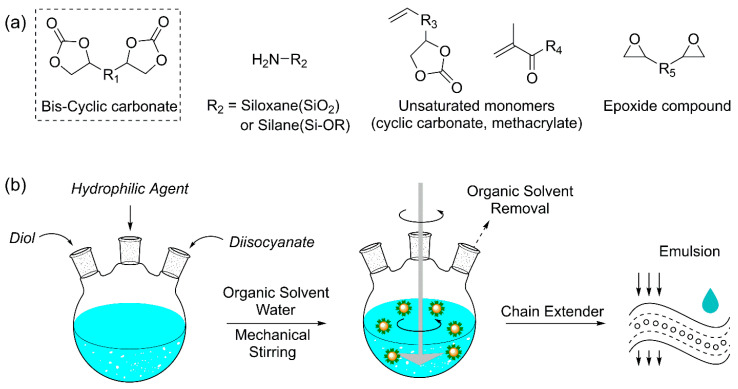
Representative building blocks of hybrid NIPUs and formation mechanisms of waterborne NIPU systems. (**a**) Chemical structures of monomers used for hybrid NIPUs, including bis-cyclic carbonates, siloxane/silane agents, unsaturated monomers, and epoxide compounds, (**b**) schematic illustration of the multi-step synthesis process for waterborne NIPU (WNIPU) emulsions.

Hybrid NIPUs are composite network structures formed by combining PHUs with other functional systems, such as epoxies, silicones, and unsaturated compounds (e.g., acrylates and polycarbonates), to address the intrinsic limitations of PHUs, including low molecular weight, high viscosity, low reactivity, and insufficient mechanical performance (Figure 10a).

The high toughness of epoxy resins, when integrated with the flexibility of NIPUs, results in enhanced mechanical properties. Although cyclic carbonates exhibit relatively low activation energies and can react under mild conditions, their slow reaction rates and limited rigidity necessitate the incorporation of epoxy compounds to overcome these drawbacks. A representative strategy involves partially carbonating epoxidized compounds with carbon dioxide to generate molecules containing both epoxide and cyclic carbonate groups. These partially carbonated epoxides subsequently react with amines to form hybrid NIPUs. By adjusting the ratio of epoxide to carbonate groups, the crosslinking density and thermomechanical properties of the resulting polymers can be precisely tuned [129]. Early studies by Rokicki et al. demonstrated that increasing the cyclic carbonate content reduced maximum exothermic heat, decreased internal stress, and improved mechanical properties, including impact resistance, shear strength, and elongation at break. Moreover, controlling this ratio enables reduced viscosity and shorter gel times during curing, thereby enhancing processability and permitting milder curing conditions [130].

Exposure to environmental factors such as ultraviolet radiation, humidity, and temperature fluctuations can compromise the durability of NIPUs. Hybrid NIPU systems incorporating unsaturated compounds provide an effective strategy to enhance environmental resistance and expand practical applicability. By introducing unsaturated monomers such as acrylates, vinyl compounds, and styrenes, NIPUs can achieve improved flexibility, weatherability, chemical resistance, and photopolymerization capability. Owing to these advantages, such hybrid NIPUs have been widely applied in adhesives, elastomers, and high-performance coatings [131].

In this approach, unsaturated cyclic carbonate monomers are first copolymerized with vinyl, acrylate, or styrene derivatives to form copolymers containing pendant carbonate groups. These side groups then undergo ring-opening aminolysis with amines to introduce β-hydroxyurethane segments, thereby forming hybrid network structures. For example, Webster and Crain synthesized copolymers based on vinyl ethylene carbonate (VEC) and reacted them with various primary amines. The resulting coatings exhibited excellent gloss, pendulum hardness, and mechanical durability [132].

The incorporation of siloxane or silane groups imparts high flexibility, hydrophobicity, and thermal stability to hybrid NIPU materials [133]. In cyclic carbonate–functionalized siloxane/silane systems, cyclic carbonate groups are first grafted onto siloxane or silane molecules, which subsequently react with diamines to form NIPU networks. The enhanced flexibility arises primarily from the presence of flexible Si–O–Si bonds and their low internal rotational barriers. Additionally, the low surface energy of siloxane segments increases polymer chain mobility, further improving the flexibility and hydrophobicity of the final materials. For example, Liu et al. synthesized silicon-based PHU materials by reacting polysiloxanes bearing cyclic carbonate side chains with diamines; the resulting polymers exhibited enhanced flexibility and hydrophobicity [134].

WNIPUs offer an environmentally friendly alternative to conventional solvent-based PU systems (Figure 10b). In applications such as paints, adhesives, and coatings, PUs have traditionally relied on volatile organic compounds (VOCs) as solvents. Replacing these with water not only mitigates environmental impact but also introduces new challenges in PU design and synthesis. A key objective in WNIPU development is the formation of stable aqueous colloidal dispersions while preserving desirable material properties [135]. Based on their stabilization mechanisms in aqueous media, WNIPUs can be classified into three categories: cationic, anionic, and nonionic. The distinct molecular design strategies associated with each type influence both dispersion stability and the performance of the resulting PU films, including water resistance, adhesion, and mechanical strength [129].

Cationic WNIPUs are synthesized through a three-step process comprising prepolymer synthesis, neutralization, and emulsification. In the prepolymer step, cyclic carbonates react with tertiary amine-containing compounds, such as N-methyldiethanolamine (NMDEA) or 3-dimethylaminopropane-1,2-diol (DMAD), to form prepolymers. These prepolymers are then neutralized using carboxylic acids, such as acetic acid, followed by the addition of deionized (DI) water to induce emulsification and yield cationic WNIPU dispersions [136,137].

Anionic WNIPUs are synthesized via a similar route, with an additional anhydride modification step introduced between neutralization and emulsification. Amine-terminated prepolymers are reacted with anhydrides such as ethylenediaminetetraacetic dianhydride (EDTAD), succinic anhydride, maleic anhydride, or o-phthalic anhydride to introduce carboxyl-terminated polymer chains. These modified prepolymers are subsequently neutralized using alkaline agents such as trimethylamine (TEA), and DI water is added to induce emulsification, producing anionic WNIPU dispersions [129].

The synthesis of nonionic WNIPUs follows a distinct three-step sequence comprising grafting modification, prepolymer synthesis, and emulsification. In the first step, hydrophilic segments are introduced into cyclic carbonates through grafting with polyols or diols such as polyethylene glycol (PEG) or its derivatives to enhance aqueous compatibility. The modified cyclic carbonates subsequently react with amine chain extenders to form nonionic WNIPU prepolymers, which are then dispersed in deionized (DI) water to generate stable WNIPU emulsions [129].

However, WNIPUs exhibit inherent limitations, including prolonged reaction times caused by restricted molecular weights and the intrinsically slow kinetics of aminolysis [138]. To address the challenges of low molecular weight and sluggish curing behavior, it is essential to develop one-pot chain extension–dispersion strategies or design reactive multifunctional cyclic carbonates.

Due to significant health concerns associated with chemical blowing agents (BAs) based on isocyanate–water reactions, research has shifted toward alternative chemistries capable of generating gaseous byproducts for the production of NIPUFs. Several of these newly developed chemical BAs can be covalently integrated into the polymer network, thereby minimizing residual byproducts within the foam structure [40]. In 2015, Cornille et al. were the first to report the synthesis of NIPUFs using poly(methylhydrogenosiloxane) as a chemical blowing agent. Foam expansion was achieved through the reaction of amines with Si–H groups on the BA, releasing dihydrogen gas [120]. The resulting foams were characterized as high-density, flexible materials with *T*_g_ ranging from −18 °C to 19 °C and thermal stability exceeding 300 °C [128]. The structural and thermal properties of these foams were influenced by the degree of crosslinking, which was governed by the functionalities of the cyclic carbonates and amine components. Although the high flammability of dihydrogen limits the industrial applicability of these NIPUFs, this study demonstrated that cyclic carbonate–amine polyaddition can be employed to fabricate both rigid and flexible NIPUFs. This finding represents a significant advancement in the development of alternative blowing agents for NIPU systems.

Overall, advances in NIPU synthesis have demonstrated significant potential to address several limitations commonly associated with conventional PU materials. Nevertheless, the production of cyclic carbonates remains in the early stages of development and has yet to be scaled for industrial application. Additionally, improved control over WNIPU synthesis is necessary to prevent hydrolysis of cyclic carbonates during polymerization. A more comprehensive understanding of the chemical polymerization kinetics in hybrid NIPU systems is also essential for further progress. Finally, challenges related to foam formation—such as the requirement for elevated temperatures or reliance on external blowing agents—must be overcome to facilitate broader industrial adoption [139].

While dynamic covalent networks and reprocessable polyurethane systems offer promising pathways toward material circularity, it should be noted that most closed-loop recycling and reprocessing strategies reported to date remain at the laboratory or proof-of-concept level. The translation of these approaches to industrial-scale implementation faces several challenges, including process complexity, energy input, and long-term material performance. Ongoing efforts toward pilot-scale validation highlight both the potential and the remaining technical barriers associated with scalable closed-loop polyurethane systems.

## 3. PU Closed-Loop Recycling from Monomer Recovery to Polymer Reprocessing

This section categorizes PU closed-loop recycling strategies into two major, complementary approaches. The first, chemical recycling, targets molecular-level closed-loop systems by selectively cleaving urethane and related bonds to recover polyols and amines (or isocyanate precursors), which can subsequently be reused for repolymerization (Section 3.1) [9]. In this context, various depolymerization pathways—including hydrolysis, acidolysis, aminolysis, and glycolysis—have been explored. Key parameters influencing process scalability include reaction selectivity, suppression of side reactions, purification efficiency, and the quality of recovered feedstocks [10].

The subsequent section focuses on recycling strategies based on dynamic covalent frameworks. Unlike complete bond scission, this approach enables reprocessability via bond-exchange reactions that rearrange the network topology while preserving the covalent backbone. By employing covalent adaptable network (CAN) designs, it becomes possible to simultaneously achieve the structural stability characteristic of thermosets and the reprocessability associated with thermoplastics (Section 3.2). In PU-CAN systems, transcarbamoylation of urethane linkages plays a central role, with either associative or dissociative mechanisms realized depending on the exchange pathway. These dynamic processes enable stress relaxation, reshaping, and ultimately extend the potential for foam-to-foam circular recycling [19].

Accordingly, this section integrates the complementary perspectives of depolymerization–repolymerization via monomer or feedstock recovery and network-preserving polymer reprocessing. In doing so, PU recycling is framed not merely as a matter of single-process optimization, but as a closed-loop platform in which material design, reaction mechanisms, and process engineering are systematically interconnected.

From a life-cycle and system-level sustainability perspective, the environmental relevance of closed-loop polyurethane recycling has been quantitatively evaluated through life cycle assessment (LCA) studies. For example, Marson et al. performed a comparative LCA of polyurethane foams produced using polyols obtained through glycolysis and those based on conventional virgin polyols [140]. Their results demonstrated that formulations containing 50–75% recycled polyol exhibited consistently lower environmental impacts across multiple categories, including climate change and fossil resource use. Specifically, reductions of approximately 15% in global warming potential and 17% in fossil resource use were reported compared with the reference virgin polyurethane foam, provided that key physical properties such as density and thermal conductivity were maintained. These findings suggest that dynamic, network-preserving recycling strategies can contribute not only to material circularity but also to measurable environmental benefits when appropriately implemented.

### 3.1. Monomer Recovery via Chemical Recycling

PUs possess three-dimensional crosslinked structures formed through urethane linkages, making thermal reprocessing significantly more challenging than for conventional thermoplastic polymers. In contrast, chemical recycling enables selective cleavage of covalent bonds in PU, allowing depolymerization into low-molecular-weight oligomers or monomeric species that can be recovered as feedstocks for subsequent PU resynthesis. Upon urethane bond cleavage, regenerated polyols can be recovered depending on the reaction pathway, while the resulting amines may be reconverted into diisocyanates via phosgene treatment. This facilitates the reacquisition of both polyols and isocyanate derivatives necessary for PU synthesis, thereby supporting the development of closed-loop PU processes. Chemical recycling encompasses a broad range of mechanisms that enable selective depolymerization tailored to specific applications. The major degradation pathways are classified based on the type of nucleophile involved in the reaction, including hydrolysis, aminolysis, and glycolysis [9,10].

#### 3.1.1. Chemical Recycling Mechanism

**Figure 11:**
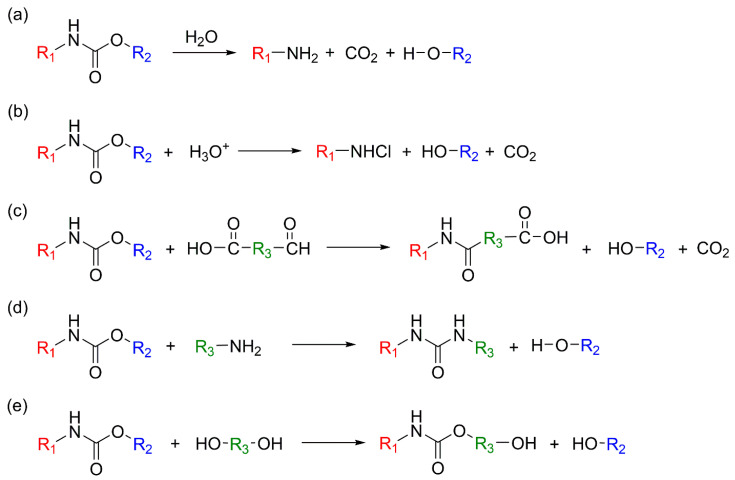
Schematic representation of major chemical recycling pathways for PU. (**a**) Hydrolysis, (**b**,**c**) acid-assisted hydrolysis (acidolysis), (**d**) aminolysis, and (**e**) glycolysis. R_1_ (red) and R_2_ (blue) represent fragments originating from the diisocyanate and polyol segments of PU, respectively, while R_3_ (green) represents the external depolymerization reagent.

Hydrolysis is one of the most fundamental and earliest investigated chemical recycling strategies for PU, involving the cleavage of urethane linkages (–NH–CO–O–) (Figure 11a). It has primarily been applied as a representative process for recovering polyols from flexible PUFs. In this reaction, water—either in liquid or vapor form—attacks the carbonyl carbon of the urethane bond, resulting in bond cleavage and the formation of amines, OH-containing compounds (polyols), and carbon dioxide (CO_2_) [15]. As such, hydrolysis constitutes a conceptually important baseline technology in PU chemical recycling, demonstrating the potential for complete material circularity through the recovery of both polyols and amines [141].

Hydrolysis is typically conducted under severe conditions, generally at temperatures of 300–400 °C and pressures of 15–50 atm. These harsh requirements lead to high energy consumption and necessitate complex processing equipment, representing major limitations to scalability. Consequently, hydrolysis has thus far remained limited to the laboratory or pilot scale, with no industrial-scale implementation reported. To overcome these challenges, acids (e.g., HCl) or bases (e.g., NaOH, NH_3_) have been introduced as catalysts. Base catalysts have been reported to improve product yields, while acid catalysts primarily enhance reaction rates [10]. In this context, urethane bond cleavage reactions originally based on water have been extended to acid-mediated depolymerization pathways, commonly referred to as acidolysis.

Acidolysis is a chemical recycling route that selectively cleaves urethane bonds in PU using organic or inorganic acids, with the reaction temperature and product characteristics strongly influenced by the type of acid employed [12]. Figure 11b illustrates acidolysis using an inorganic acid (HCl), wherein urethane bonds react with HCl to produce amine salts (R_1_–NHCl), polyols (HO–R_2_), and CO_2_. This HCl-based acidolysis is typically conducted at relatively mild temperatures around 60 °C. However, as the resulting amines are produced in salt form, additional purification steps are required.

Figure 11c illustrates acidolysis using organic dicarboxylic acids (DCAs), wherein urethane bonds react with DCAs to generate amide-based degradation products (R_1_–NH–CO–R_3_–COOH), polyols (HO–R_2_), and CO_2_. Unlike the amine salts formed in HCl-based acidolysis, DCA-derived amide products can be directly utilized as functional materials, such as adhesives, thereby broadening the applicability and enhancing the overall efficiency of the acidolysis process. Organic acid-based acidolysis generally yields recycled polyols with relatively low OH values, making them suitable for the production of flexible PUFs. Reaction temperature—typically 190–210 °C—and the PU/DCA ratio are key parameters governing the properties of the recovered polyols [12,14,142]. These reactions are commonly conducted under inert atmospheres to suppress side reactions at elevated temperatures. Although many acidolysis processes proceed under catalyst-free or self-catalyzed conditions, catalysts such as AlCl_3_, ZrO_2_, WO_3_, and WO_3_–ZrO_2_ have been reported to enhance both reaction rates and efficiencies [12].

Recent studies have also focused on improving the applicability of hydrolysis by elucidating underlying reaction mechanisms and establishing structure–reactivity relationships. Motokucho et al. reported that introducing CO_2_ into the system maintains mildly acidic conditions (pH 3.5–4.0) due to the formation of carbonic acid, thereby promoting selective urethane bond cleavage. Subsequent FT-IR, ^1^H NMR, and GPC analyses confirmed that the polymeric residues remained as PU oligomers with reduced molecular weights, while other chemical bonds were largely preserved. Additionally, investigation of CO_2_ pressure effects revealed that increased acidity accelerated PU degradation, although pressures above 5.9 atm had minimal further influence on the degradation rate. Based on these findings, a hydrolysis mechanism for PU in the presence of CO_2_ was proposed [143,144].

In parallel, the hydrolytic resistance of PU has been shown to strongly depend on the structural characteristics of the monomers, including substituent size, side-chain density, and carbon number. Model PU studies by Murata et al. demonstrated that PUs derived from polyols with longer carbon chains or greater steric hindrance exhibited enhanced hydrolytic resistance, whereas PUs based on 1,4-butanediol (BD) degraded most rapidly. These findings offer critical design guidelines for predicting PU stability in water-contact environments.

To address the challenges associated with high temperature and pressure, a hydroglycolysis approach using mixtures of water and glycerol (or sorbitol/glycerol) has been proposed. This method, which combines hydrolysis and glycolysis, enables PU degradation under comparatively milder conditions at approximately 200 °C and, in some cases, is further facilitated by microwave irradiation [145]. However, similar to conventional hydrolysis, hydroglycolysis requires the complete separation of polyols and amines, necessitating solvent extraction and other complex purification steps, which remain substantial barriers to industrial application [10,19].

Building on the acidolysis mechanisms and characteristics discussed above, recent studies have focused on optimizing practical reaction conditions for DCA-based acidolysis. Gama et al. systematically investigated the effects of key parameters—such as temperature, reaction time, and PU/DCA ratio—using SA and phthalic acid, and demonstrated that these variables significantly influence the properties of the recovered repolyols, including OH value, acid value (AV), and viscosity. In particular, SA produced repolyols with higher OH values and more complete depolymerization compared to phthalic acid, resulting in improved yields. However, DCA-based acidolysis involves competing mechanisms at elevated temperatures, where thermal degradation of PU can occur simultaneously. Under these conditions, isocyanates and amines may be generated and subsequently react with DCAs to form amides, releasing CO_2_ and water as byproducts. The repolyols obtained via acidolysis were applicable in PUF formulations at inclusion levels of up to 30%; nevertheless, reductions in mechanical properties, including modulus, compressive strength, and toughness, were observed. As a result, their application was considered more suitable at additive levels rather than as full polyol replacements. Additionally, due to the formation of repolyol–DCA ester structures, a deficiency of free OH groups was reported, necessitating further purification steps and the incorporation of short-chain diols (e.g., ethylene glycol or propylene glycol) to reintroduce terminal OH functionalities [13,14,144]. Overall, DCA-based acidolysis exhibits considerable potential for repolyol recovery and the realization of closed-loop PU recycling. Nonetheless, addressing challenges such as competing thermal degradation reactions, byproduct formation, and insufficient free OH groups requires precise control over reaction conditions and the development of effective purification strategies, which are essential for successful industrial implementation.

Aminolysis is a depolymerization reaction in which amines act as nucleophiles to cleave urethane linkages (Figure 11d). In this process, amines attack the carbonyl carbon of the urethane bond, resulting in the formation of urea-based degradation products and polyols. Owing to their relatively high molecular weight, these degradation products readily undergo phase separation: the upper phase is enriched in high-purity polyols with elevated OH values, making it suitable for flexible PU production, whereas the lower phase contains urea byproducts and heavy oligomers with lower OH values, which are more appropriate for rigid PU applications [15,16]. Aminolysis can proceed under relatively mild conditions (80–190 °C) and offers high selectivity with a comparatively low yield of undesired byproducts. However, the resulting reaction mixture is highly complex, containing polyols, urea-based byproducts, urethane fragments, and residual amines. In particular, unreacted primary and secondary amines exhibit substantially higher reactivity toward isocyanates than the alcohols generated during depolymerization, leading to the rapid formation of urea linkages during subsequent PU synthesis. This competitive reaction interferes with PU re-synthesis using recycled polyols, ultimately resulting in inferior material performance. Moreover, excessive aminolysis increases the concentration of low-molecular-weight species and urea groups, thereby reducing solubility and phase stability, while intensifying the demands of purification and separation. As a result, aminolysis generally does not yield polyols of sufficient purity for use as primary components in new PU formulations; instead, the recovered polyols are typically employed in limited quantities, such as low-content additives [12].

To address these limitations, recent studies have focused on improving the selectivity of aminolysis conditions and controlling product composition. Xue et al. evaluated the effects of reaction temperature and amine structure on aminolysis kinetics and product viscosity using various aliphatic polyamines, including DETA, TETA, and TEPA. Their results demonstrated that aminolysis is highly dependent on both the amine structure and reaction temperature, and that appropriate selection of reaction conditions can enable rapid depolymerization while maintaining acceptable polyol quality [146]. Chuayjuljit et al. investigated PU aminolysis at 180 °C using DETA, with NaOH added to the reaction system. Increasing NaOH concentration decreased product viscosity and increased amine content, indicating enhanced PU depolymerization. The authors proposed that NaOH functions not only as a catalyst but also as a depolymerization reagent capable of cleaving both urethane and urea bonds. This interpretation aligns with findings by Van der Wal, who demonstrated urethane bond cleavage using KOH and alkanolamines. In such systems, metal hydroxides cleave urethane linkages to form primary amines, polyols, and metal carbonates, whereas reactions involving urea bonds produce two primary amines without polyol formation [147]. Overall, aminolysis remains a promising pathway due to its efficiency in PU depolymerization; however, further chemical and process-level optimizations are required to address challenges related to product quality and the complexity of separation and purification.

Glycolysis is the most extensively studied and industrially mature chemical recycling process for PU (Figure 11e). In this reaction, glycols such as ethylene glycol (EG) and diethylene glycol (DEG) nucleophilically attack the carbonyl carbon of urethane linkages, thereby inducing transesterification that yields glycolyzed products comprising regenerated polyols and urethane-containing fragments [17,18,148]. The process is typically carried out at approximately 200 °C, and the use of catalysts—such as acetates, alkali metal hydroxides, or tin-based organometallic compounds—enables high depolymerization efficiency within relatively short reaction times. At temperatures below 180 °C, the reaction rate decreases significantly, whereas temperatures above 220 °C initiate thermal degradation of urethane linkages, thereby increasing amine formation. As such, precise temperature control is considered a critical parameter in glycolysis. The composition of glycolysis products is strongly influenced by the type of PU waste (rigid or flexible), the glycol employed, and the PU/glycol ratio. During the reaction, free amines are generated, which can react rapidly with isocyanates, leading to excessive crosslinking and compromising the mechanical properties of resynthesized PU materials. In particular, aromatic amines require stringent concentration control due to their toxicity. Although using a large molar excess of glycol enhances depolymerization efficiency, it also increases process costs and complicates downstream purification, presenting a significant practical limitation [10]. The reactivity of glycolysis is highly dependent on the glycol structure and the catalyst employed. Short-chain glycols provide greater accessibility to urethane linkages, enabling faster depolymerization, whereas DEG exhibits slower reactivity but yields higher-viscosity products and improved phase separation, which can facilitate purification. Common catalysts include NaOH, potassium acetate (KOAc), FeCl_3_, tin-based compounds, and selected ionic liquids [149,150,151,152]. These catalysts promote transesterification either by forming metal alkoxides through reaction with glycols or by directly activating carbamate bonds, thereby reducing depolymerization time and allowing control over the OH value of the recovered polyols.

Recent studies have expanded glycolysis research by focusing on improving reaction efficiency and product selectivity. Heiran et al. investigated various metal catalysts and reported that metal hydroxides and octoate catalysts activate urethane carbonyls through metal alkoxide formation, whereas metal acetates directly activate carbamate bonds via distinct reaction pathways. These findings suggest that catalyst basicity and the stability of metal–carbonyl coordination are critical determinants of depolymerization behavior [153,154]. Johansen et al. demonstrated that tert-amyl alcohol can depolymerize both flexible PU and post-consumer PU waste, achieving over 95% recovery of toluene diamine (TDA) and quantitative recovery of repolyols under KOH-catalyzed conditions at 225 °C. These results underscore the potential of glycolysis to evolve into a closed-loop recycling route capable of recovering not only polyols but also isocyanate precursors [155]. Overall, glycolysis proceeds rapidly under relatively mild conditions and facilitates the recovery of high-purity, reusable products, making it one of the most practical PU recycling technologies currently available. Nonetheless, challenges persist in controlling amine formation, managing the process economics associated with excess glycol usage, and optimizing conditions for different PU waste streams. Recent advances in catalyst design, reaction acceleration strategies, and the development of novel depolymerization agents continue to enhance the industrial feasibility of glycolysis-based PU recycling [10,12].

#### 3.1.2. Process Intensification and Advanced Approaches

Although the individual depolymerization pathways discussed above offer effective strategies for selectively cleaving urethane linkages in PU, their implementation in practical recycling processes remains constrained by challenges such as suppression of side reactions, complex purification requirements, and deterioration in the quality of recovered feedstocks. These limitations underscore the need for process intensification strategies that transcend conventional depolymerization-based approaches, aiming instead at the recovery of high-purity polyols and isocyanate precursors. In this context, a series of recent studies has reported significant advances intended to enhance the selectivity, controllability, and overall circularity of PU chemical recycling.

**Figure 12:**
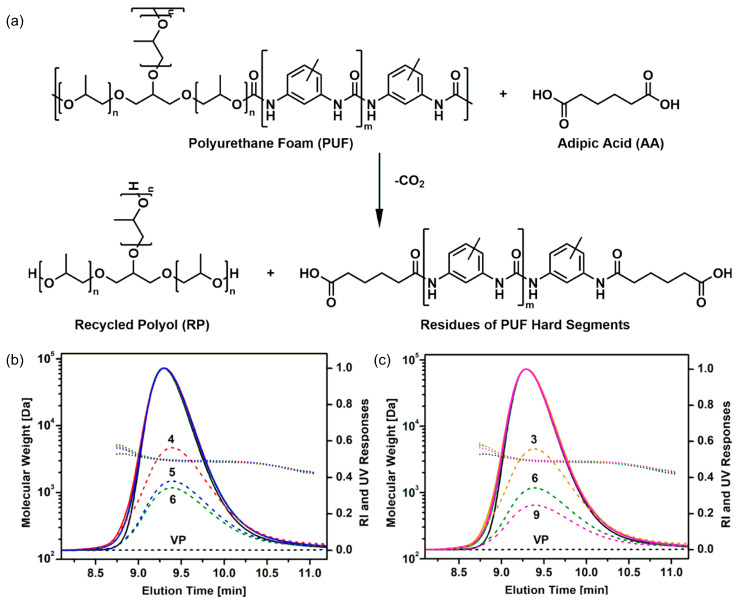
Microwave-assisted acidolysis and aminolysis of polyurethane foam for efficient closed-loop polyol recovery. (**a**) Reaction scheme of MW-assisted acidolysis of polyurethane foam (PUF) using adipic acid, illustrating selective cleavage of urethane linkages to yield recycled polyol and hard-segment residues with CO_2_ evolution; (**b**,**c**) size-exclusion chromatography (SEC) traces of recovered polyols after MW-assisted acidolysis and aminolysis, respectively, showing molecular weight distribution and dispersity comparable to those of virgin polyol. The results demonstrate rapid depolymerization, effective phase separation, and preservation of polyol quality under microwave-enhanced conditions, supporting the feasibility of closed-loop polyurethane recycling. Reprinted from [156] under CC-BY 4.0 License.

Žagar and co-workers applied microwave(MW)-assisted process intensification to systematically address the slow reaction kinetics, purification complexity, and polyol quality degradation frequently encountered in PU acidolysis and aminolysis (Figure 12). In their MW-assisted acidolysis study, AA was employed as the depolymerizing agent, and reactions conducted at 210–230 °C achieved near-complete depolymerization within 15–40 min, a substantial improvement over conventional acidolysis processes, which typically require several hours. Following the reaction, products spontaneously separated into a polyol-rich phase and a hard-segment residue. Low-molecular-weight amines, including toluene diamine (TDA), were effectively removed via liquid–liquid extraction using ethyl acetate (EtOAc) and 0.1 M HCl–water. The purified polyol exhibited molecular weight (Mw) and dispersity (Đ) comparable to those of virgin polyols and was fully suitable for flexible foam production at 100% replacement levels. Increasing the acid loading enhanced urethane bond cleavage selectivity and reduced TDA formation, while also promoting esterification of polyol end groups, revealing a trade-off between depolymerization selectivity and end-group modification. Notably, repeated acidolysis cycles using the same recovered polyol did not result in significant changes in molecular weight or end-group composition, thereby experimentally confirming the feasibility of closed-loop recycling based on acidolysis [156].

In a subsequent study, the same MW-assisted strategy was extended to amine-based depolymerization to address the inherent limitations of conventional aminolysis. Utilizing highly reactive polyamines such as tris(2-aminoethyl)amine (TREN) and polyethyleneimine (PEI), reactions conducted at approximately 220 °C achieved selective cleavage of more than 99% of urethane bonds. Common drawbacks associated with aminolysis—including excessive urea accumulation, the formation of highly viscous mixtures, and challenges in phase separation and purification—were substantially mitigated. Following depolymerization, the polyol-rich phase was isolated and subjected to a sequential purification protocol comprising EtOAc extraction, acid treatment, water washing, and low-temperature vacuum evaporation. This process effectively removed residual amines and low-molecular-weight byproducts. The recovered polyols exhibited molecular weight, dispersity, and purity comparable to those of virgin materials. Soft foams produced using 100% of these recycled polyols showed no deterioration in mechanical properties, confirming that aminolysis-derived polyols can be fully reutilized in new PU formulations [157]. Collectively, these studies by Žagar and co-workers demonstrate a process-optimized recycling paradigm that meets critical requirements for high-purity polyol recovery, short reaction times, reduced purification complexity, preserved material quality, and experimentally validated closed-loop feasibility. This body of work marks a paradigmatic shift in PU chemical recycling, advancing the field from basic depolymerization toward an integrated platform that simultaneously addresses process design, reaction selectivity, material performance, and circularity.

**Figure 13:**
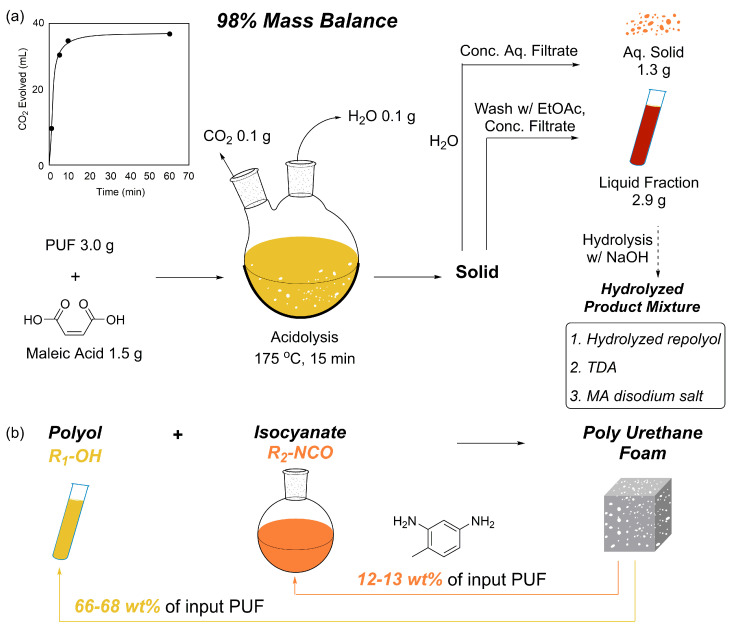
Solvent-free acidolysis of PUF using maleic acid enables rapid repolyol recovery and generation of isocyanate precursors. (**a**) Schematic illustration of maleic acid–based depolymerization under solvent-free conditions, highlighting accelerated breakdown of PUF, near-quantitative mass balance, and CO_2_ evolution; (**b**) material flow and mass balance of the process, including phase separation, aqueous workup, and alkaline hydrolysis, resulting in high repolyol recovery (66–68 wt% of input PUF) and toluene diamine (TDA) yield (12–13 wt%), demonstrating the broader potential of acidolysis beyond polyol reclamation.

Christopher and co-workers established structure–reactivity correlations for polyol recovery via acidolysis and, based on these insights, proposed an advanced acidolysis process model with clear potential for industrial scale-up (Figure 13a). While earlier work by Gama and co-workers demonstrated the feasibility of repolyol production from dicarboxylic acids (DCAs), the process remained limited in reaction rate and selectivity. In contrast, Christopher et al. significantly improved the time and energy efficiency of acidolysis by employing a solvent-free maleic acid-based system, achieving polyol recovery exceeding 95% in under 10 min at approximately 175 °C. Furthermore, mechanical size reduction of the PUF before reaction maximized the contact area between the foam and maleic acid, dramatically enhancing reaction kinetics. These findings indicate that acidolysis is influenced not only by chemical reaction conditions but also by mass transport and interfacial surface area, underscoring its nature as a process-sensitive depolymerization pathway. In terms of product distribution, approximately 66–68% of high-purity repolyol and 12–13% of toluene diamine (TDA) were consistently recovered relative to the input PUF, corresponding to an overall material recovery of around 80% (Figure 13b). Importantly, these results demonstrate that acidolysis-based depolymerization extends beyond polyol recovery, offering a viable route for the recovery of isocyanate precursors such as TDA and emphasizing its significant process-level implications for closed-loop PU recycling [158].

**Figure 14:**
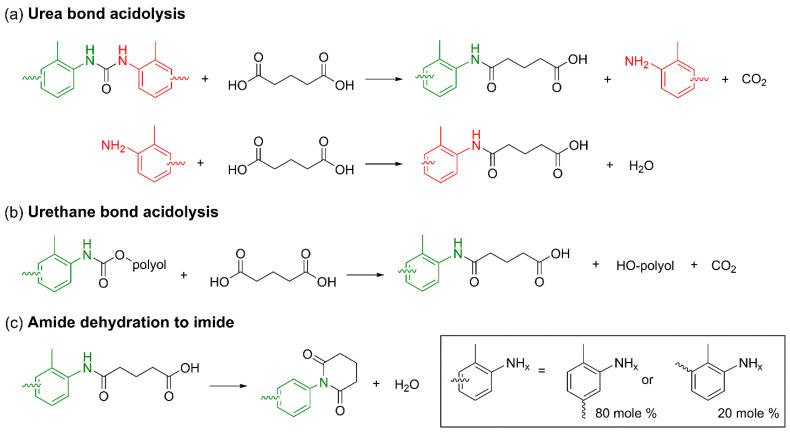
Reaction pathways of dicarboxylic-acid-mediated acidolysis of PU. (**a**) Acidolysis of a urea bond yields an amide, amine, and CO_2_ (top), with the amine intermediate further converted to an amide in the presence of excess dicarboxylic acid (bottom); (**b**) acidolysis of a urethane bond produces an amide, polyol, and CO_2_, and (**c**) intramolecular dehydration of the amide intermediate leads to imide formation.

Subsequently, Christopher and co-workers elucidated that the structural characteristics of dicarboxylic acids (DCAs) are key determinants governing both the formation of nitrogen-containing byproducts and the cleavage pathways of urethane bonds, thereby establishing a structure–selectivity framework for acidolysis (Figure 14). In particular, the distance between carboxyl groups and the conformational flexibility of the DCA backbone were shown to exert a dominant influence on the reaction pathways. SA and phthalic acid (PiA) possess identical carboxyl group separations but differ significantly in structural flexibility. SA, with its aliphatic and flexible backbone, enables the initially formed amide intermediates to readily fold and undergo rapid intramolecular cyclization, yielding thermodynamically stable imide species within 2–3 h. In practical maleic PUF (M-PUF) acidolysis experiments, cleavage of urethane and urea linkages was completed within approximately 10–25 min, and the nitrogen-containing intermediates were predominantly converted into imides. By contrast, PiA exhibits a rigid aromatic backbone that restricts chain mobility and intrinsically disfavors amide-to-imide conversion. Nevertheless, due to its similarly short carboxyl group separation relative to SA, rapid cyclization still occurs, yielding overall reaction behavior comparable to that of SA. Notably, glutaric acid (GA), which contains one additional methylene unit compared to SA, exhibited markedly different behavior. The extended –CH_2_– backbone of GA hinders the molecular folding required for efficient intramolecular cyclization, thereby delaying direct conversion from amide to imide species. In GA-based acidolysis, urethane bond cleavage proceeded rapidly within 20–60 min; however, diamide species persisted in the reaction mixture for 8–24 h, and amide–imide hybrid intermediates remained at high concentrations for more than 24h, indicating that imide formation constituted a kinetically slow step. Consequently, GA systems displayed a stepwise transformation sequence—from amide to amide–imide hybrids and eventually to imide—accompanied by significant accumulation of intermediates. These findings clearly demonstrate that DCA structural parameters—including carboxyl group separation, backbone flexibility, and aromatic rigidity—are critical design variables for controlling depolymerization selectivity. This structure–reactivity framework subsequently provided the foundation for strategies aimed at tuning product distributions via acid loading control and for further optimization of acidolysis-based recycling processes [159].

Subsequent kinetic studies employed the shrinking core model (SCM) to quantitatively elucidate differences in rate-controlling mechanisms as a function of acid structure and phase behavior. For GA, AA, and PiA, which exist in the liquid phase under reaction conditions, depolymerization proceeded under reaction-controlled regimes. In contrast, SA, which initiates the reaction in the solid state, exhibited film-diffusion-controlled behavior, where mass transport through a liquid boundary layer governed the overall reaction rate. Notably, in all systems, depolymerization commenced at relatively low temperatures (~165 °C), strongly supporting a DCA-based direct scission mechanism that is fundamentally distinct from conventional high-temperature thermal degradation pathways. At high conversion levels, transitions in the dominant reaction mechanism were observed, driven by strut collapse, increased viscosity, and concentration changes in the reaction medium. Collectively, these findings establish an integrated kinetic–mechanistic framework capable of predicting the dominance and interplay of depolymerization pathways as a function of key process variables, including acid structure, phase behavior, acid loading, and temperature. This framework advances chemical recycling from a strategy focused solely on accelerating depolymerization rates to a process-engineering platform in which product selectivity and recovery efficiency can be deliberately designed. Importantly, it provides a critical foundation for the practical industrial implementation of acidolysis-based PU recycling, encompassing scale-up, reactor design, and optimization of acid feeding strategies [160].

Conventional chemical recycling approaches for PU have predominantly focused on polyol recovery, whereas direct regeneration of isocyanates (–NCO) remains impractical due to multiple chemical and process-related barriers. These include the intrinsic reactivity of isocyanates, their rapid decomposition at elevated temperatures, and immediate secondary reactions following formation. In particular, the fact that most depolymerization processes are conducted at high temperatures (>180 °C), in the presence of moisture, or under basic conditions render preservation of NCO species nearly impossible. As a result, prior studies have largely converged on pathways that fully decompose urethane linkages into amines and polyols, rather than pursuing direct isocyanate recovery.

**Figure 15:**
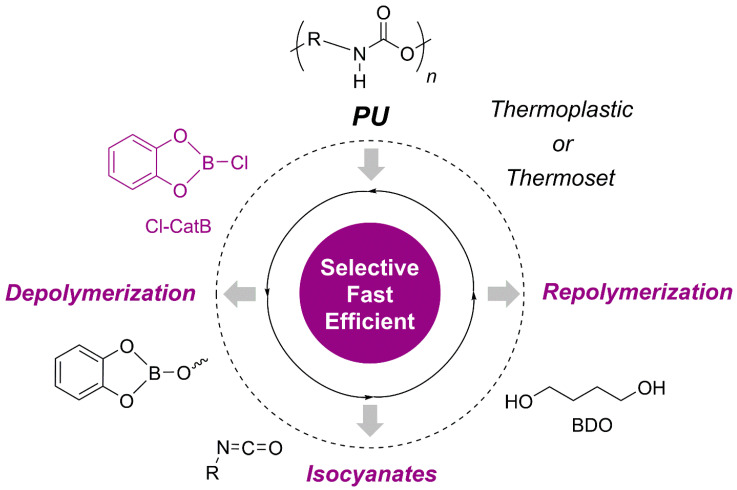
Selective closed-loop depolymerization and repolymerization of PUs via Cl-CatB-mediated retro-carbamoylation, enabling direct recovery of isocyanates.

O’Dea and co-workers addressed these longstanding limitations by introducing a novel depolymerization mechanism based on *β*-chlorocatecholborane (Cl-CatB), enabling the direct recovery of isocyanates from PU (Figure 15). The key feature of this approach is the Lewis-acidic organoboron character of Cl-CatB, which selectively cleaves urethane linkages via a retro-carbamoylation pathway rather than via hydrolysis. Specifically, Cl-CatB activates the carbonyl oxygen of the urethane bond through Lewis acid coordination, promoting selective bond cleavage and releasing the corresponding isocyanate. Simultaneously, the liberated alcohol is trapped by the borane to form a stable alcohol–borane adduct, effectively suppressing secondary reactions between free alcohols and isocyanates that would otherwise lead to urethane reformation. Importantly, the reaction proceeds under mild conditions (<100 °C) in tetrahydrofuran (THF) with triethylamine, thereby avoiding the thermal decomposition of isocyanates commonly encountered in conventional thermolytic processes. This reaction environment exhibits sufficiently high selectivity to achieve quantitative cleavage of urethane bonds in both thermoplastic and thermoset PU systems. As a result, MDI and TDI were recovered in high yields (85–100%), and subsequent repolymerization experiments demonstrated thermal and molecular-weight characteristics comparable to those of the virgin materials, confirming the complete preservation of isocyanate structural integrity. This work represents a significant advancement beyond the fundamental limitations of conventional PU recycling paradigms by establishing a phosgene-free route for isocyanate recovery directly from PU waste. Nevertheless, challenges remain regarding industrial scalability, including the cost and safety concerns associated with specialized boron reagents such as Cl-CatB, the use of organic solvents, and the reduced yields observed for commercial PU waste streams. These issues underscore the need for further process optimization and improvements in reagent efficiency. Despite these challenges, this approach marks a pivotal transition, elevating isocyanate recovery from a conceptual possibility to a process-relevant and technologically viable recycling strategy [161].

### 3.2. Dynamic Covalent Frameworks for Thermoset PU Recycling

The chemical recycling strategies discussed above are based on the selective cleavage of urethane bonds in PU to recover low-molecular-weight polyols and amines, which can subsequently be converted back into isocyanate and polyol feedstocks for PU synthesis. These approaches rely on the irreversible scission of covalent bonds and enable ideal closed-loop processes through molecular-level feedstock recovery. However, they continue to face challenges associated with harsh operating conditions, including elevated temperatures and pressures, the use of additional reagents, process complexity, and high energy consumption. As an alternative paradigm, increasing attention has recently been directed toward dynamic covalent framework concepts that enable the reprocessing and recycling of thermoset PU without complete covalent bond cleavage. This approach is based on the incorporation of reversibly exchangeable urethane linkages, allowing polymer networks to be reshaped or rearranged while preserving their crosslinked architecture. Polymer systems operating under this principle are classified as CANs, which fundamentally differ from conventional depolymerization-based recycling by combining the mechanical stability of thermosets with the processability of thermoplastics [9]. In PU-based CANs, the key enabling reaction is transcarbamoylation of urethane bonds. Depending on the exchange pathway, this reaction can proceed via an associative mechanism, wherein the network crosslink density is preserved throughout bond exchange, or a dissociative mechanism, which involves temporary cleavage and reformation of urethane bonds [19]. These mechanisms exhibit intrinsically different characteristics in terms of rheological behavior, reprocessing temperature, network stability, and suitability for circular recycling. Accordingly, the design of sustainable PU recycling strategies requires not only consideration of whether urethane bonds are cleaved, but also a clear understanding of the operative mechanisms governing urethane bond exchange.

#### 3.2.1. Dynamic Urethane Exchange in PU CANs: Associative and Dissociative Mechanisms

Polymers are broadly classified into thermoplastics and thermosets. Thermoplastic polymers consist of linear or branched chains with finite molecular weights, exhibiting reversible softening upon heating and rapid solidification upon cooling. Due to this reversible thermal behavior, thermoplastics offer excellent processability and reprocessability; however, they generally suffer from limited dimensional stability and poor creep resistance. In contrast, thermosetting polymers form three-dimensional crosslinked networks corresponding to effectively infinite molecular weight. These materials are typically processed as low-viscosity precursors that undergo curing reactions, enabling favorable flow characteristics for the fabrication of structural composites but resulting in slower processing rates. Thermosets exhibit superior dimensional stability and creep resistance; however, the permanent covalent crosslinks that impart these properties also render them extremely difficult to reprocess or recycle, thereby contributing significantly to plastic waste and raising critical sustainability concerns [9,162,163]. As the recycling of thermosetting plastics has become an increasingly important objective in the plastics industry, corresponding regulatory frameworks have begun to emerge. In this context, CANs have been proposed as a promising strategy for developing sustainable plastics. CANs are polymer architectures that maintain a three-dimensional covalent network while enabling dynamic bond exchange under thermal and/or catalytic conditions. This dynamic behavior allows for the simultaneous realization of the mechanical strength, thermal resistance, and chemical stability characteristic of thermosets, along with the reprocessability and adaptability typically associated with thermoplastics [162,164].

**Figure 16:**
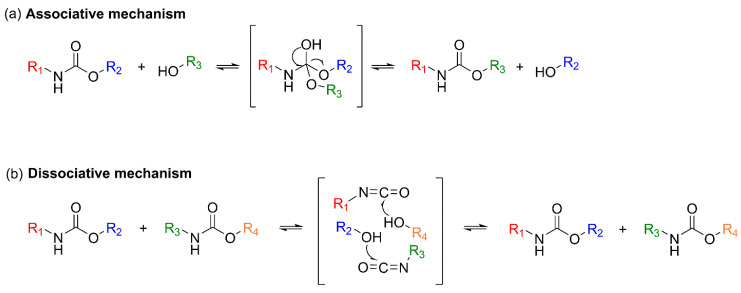
(**a**) Associative exchange mechanism of urethane bonds proceeding via nucleophilic substitution, (**b**) dissociative exchange mechanism involving isocyanate intermediates.

PU is a representative thermosetting polymer extensively utilized across numerous industrial sectors. However, once cured, PU forms a permanently crosslinked three-dimensional network, which imposes inherent limitations on recyclability and reprocessability, thereby contributing to disposal challenges and environmental burdens [163,165,166,167]. As a result, research on PU-based CANs has expanded rapidly since approximately 2010. CANs are typically designed such that crosslinking bonds undergo dynamic exchange at elevated temperatures in the presence of catalysts and are classified as either associative or dissociative, depending on the bond exchange mechanism (Figure 16). In PU-based systems, transcarbamoylation (TC) is the most critical dynamic bond exchange reaction, proceeding in the presence of free OH groups via two distinct pathways. The associative mechanism follows an addition–elimination sequence analogous to transesterification, while the dissociative mechanism involves temporary cleavage of urethane bonds into alcohol and isocyanate species, followed by recombination [168,169,170].

The associative mechanism is a concerted bond-exchange process in which new bonds form prior to the cleavage of existing ones, thereby preserving the overall crosslink density throughout the exchange reaction [171]. When bond exchange is activated at elevated temperatures, the network topology evolves progressively, resulting in distinctive rheological behavior characterized by Arrhenius-type viscosity dependence. A key parameter governing this behavior is the topology freezing transition temperature (*T*_v_), defined as the temperature at which the network viscosity reaches approximately 10^12^ Pa·s. Depending on the relative positions of the *T*_g_ and *T*_v_, the network can transition from a glassy or rubbery solid to a viscoelastic liquid state. This thermally activated dynamic behavior enables various functionalities, including stress relaxation, self-healing, welding reprocessing, and shape-memory effects. Vitrimers represent the prototypical implementation of associative CANs, wherein the total number of covalent bonds in the network remains constant during bond exchange, while the network topology is continuously rearranged. As a result, effective stress relaxation occurs at elevated temperatures. In 2011, Leibler and co-workers introduced the concept of vitrimers and demonstrated that activating bond exchange at elevated temperatures induces gradual network topology rearrangement, accompanied by an Arrhenius-type decrease in viscosity with increasing temperature [170,172,173]. To implement the associative mechanism in practical polymer networks, various exchange reactions—such as transcarbamoylation and transesterification—have since been developed. Early studies on CANs primarily aimed to address the intrinsic limitations of damage recovery in thermosetting resins, which stem from restricted chain mobility and fixed crack interfaces. The introduction of associative CANs based on dynamic bond exchange enabled network rearrangement, facilitating repeated crack healing and damage recovery. As a result, extensive research has focused on the self-healing and welding of polymeric materials through CAN architectures. These initial efforts sought to enable damage recovery in PU by leveraging stress relaxation and partial structural reorganization, particularly through the rejoining of fractured interfaces via network-level bond exchange. However, this early stage of research represented only a preliminary exploration and did not fully exploit the broader capabilities of transcarbamoylation [168]. More recently, Fortman and co-workers reported a polyhydroxyurethane (PHU)-based vitrimer in which an associative transcarbamoylation pathway was experimentally shown to operate stably even in the absence of external catalysts. This discovery marked a pivotal advancement in TC-based dynamic PU research, expanding its scope beyond self-healing to include reprocessing and reshaping of thermoset PU networks, and ultimately enabling circular recovery of end-of-life PU materials.

**Figure 17:**
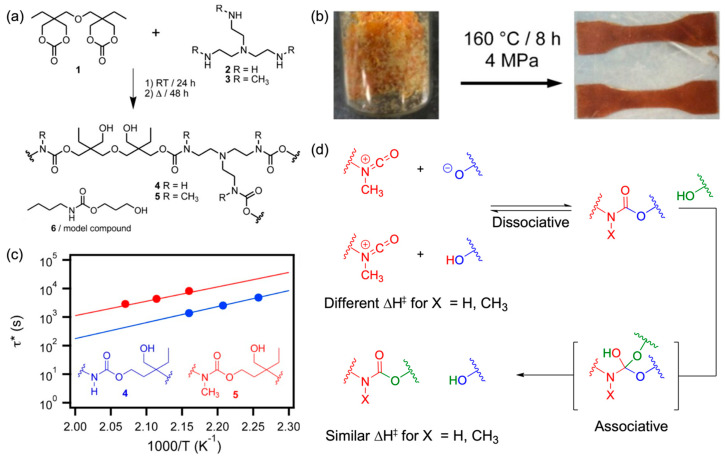
Catalyst−free associative transcarbamoylation in polyhydroxyurethane (PHU) vitrimer networks enabling reprocessability and stress relaxation. (**a**) Synthesis of PHU networks via polyaddition between bis(6−membered cyclic carbonate) monomers and trifunctional amines, forming hydroxyurethane linkages with pendant hydroxyl groups; (**b**) representative photographs demonstrating thermal reprocessing and reshaping of the PHU vitrimer under hot−pressing conditions (160 °C, 8 h, 4 MPa), evidencing vitrimer−like malleability without network degradation; (**c**) Arrhenius plots of stress relaxation times (τ*) for PHU networks with different N−substituents (X = H, CH_3_), showing tunable relaxation kinetics while maintaining associative exchange behavior; and (**d**) schematic comparison of dissociative and associative transcarbamoylation pathways. In PHU vitrimers, neighboring hydroxyl groups nucleophilically attack carbamate linkages to form orthocarbamate intermediates, followed by alkoxy exchange without bond dissociation, resulting in a purely associative exchange mechanism. The similar activation enthalpies (ΔH^‡^) for X = H and CH_3_ further support the associative nature of the exchange reaction. Adapted with permission from Ref. [174]. Copyright 2015 American Chemical Society. Open Access under the ACS Author Choice License.

Fortman and co-workers first reported in 2015 that a PHU network, formed via the reaction of a bis(6-membered cyclic carbonate) with a trifunctional amine, functions as a urethane vitrimer. (Figure 17) This material exhibits pronounced stress relaxation and reprocessability at elevated temperatures without the need for external catalysts. In this network, free OH groups located adjacent to the carbamate linkages nucleophilically attack the carbamate moiety, forming an orthocarbamate intermediate, followed by the elimination of an alkoxy group (–OR) and regeneration of the carbamate linkage. The process proceeds via an addition–elimination-based transcarbamoylation pathway, characteristic of an associative exchange mechanism. FT-IR analysis revealed no emergence of new functional groups before or after the stress relaxation experiments, confirming that network rearrangement occurs through a purely associative mechanism without bond dissociation. Furthermore, the activation energy for stress relaxation of the PHU vitrimer (*E*_a_ = 111 ± 10 kJ·mol^−1^) was significantly lower than that of the corresponding molecular model system measured under identical conditions (*E*_a_ = 148 ± 7 kJ·mol^−1^). Density functional theory (DFT) calculations attributed this discrepancy to a mechanochemical activation effect, whereby mechanical deformation (i.e., chain tension) induces twisting of the N–C(O) π-conjugation, thereby facilitating transcarbamoylation and accelerating the exchange reaction [174].

Subsequent studies further elucidated the influence of monomer structure on the thermal stability and exchange kinetics of PHU vitrimers. In particular, PHUs derived from 5-membered cyclic carbonates (5CCs) were shown to undergo rapid reverse reactions at 180 °C, wherein hydroxyurethane linkages revert to cyclic carbonates and amines. The released amines subsequently react with the remaining carbonate groups, leading to decarboxylation and the formation of undesired byproducts, indicating competing thermal degradation pathways that preclude vitrimer behavior. In contrast, PHUs based on 6-membered cyclic carbonates (6CCs) exhibited no detectable network degradation or reverse conversion under identical conditions. Instead, these systems maintained structural integrity while enabling dynamic associative exchange. Depending on the polymer backbone architecture (e.g., oligoether versus aliphatic chains), crosslink density, and the accessibility of OH groups adjacent to carbamate linkages, the activation energy for stress relaxation could be systematically tuned within the range of approximately 99–136 kJ·mol^−1^. These structure–reactivity correlations highlight the critical roles of carbonate ring size, backbone flexibility, and crosslinking motifs in the rational design of urethane-based associative CANs. Moreover, 6CC-based PHU vitrimers recovered approximately 70–80% of their original tensile strength and elastic modulus after reprocessing, demonstrating that such systems extend beyond self-healing materials to constitute a viable platform for the reshaping and reprocessing of thermoset PUs [175].

Subsequently, the concept of associative urethane exchange has been extended to enable circular recycling of post-consumer PUs. Recent studies on thermoset PU foam-to-foam reprocessing have demonstrated that conventional crosslinked PUFs can be converted into dynamic networks through catalyst-mediated carbamate exchange, thereby allowing thermomechanical melt reprocessing and refoaming under twin-screw extrusion conditions. The regenerated foams exhibited bulk density, cellular morphology, and compressive properties comparable to those of the original materials, while maintaining structural integrity even after multiple reprocessing cycles. These findings indicate that associative urethane exchange offers a practical mechanism for closed-loop polymer recovery by enabling direct reuse of the polymer network, rather than relying solely on molecular depolymerization and repolymerization. Collectively, these advances demonstrate that transcarbamoylation-based associative exchange has evolved beyond its initial focus on crack healing and welding and is now emerging as a viable macroscopic sustainability strategy for thermoset PUs, encompassing reprocessing, reforming, and foam-to-foam circular recycling [176].

In principle, both dissociative and associative mechanisms are feasible pathways for urethane exchange. However, multiple studies have suggested that, given the intrinsic structure of conventional PUs and the typical activation conditions employed, urethane exchange predominantly proceeds via a dissociative mechanism. Indeed, in most reported polyether- and polyester-based PU-CAN systems to date, dissociative urethane exchange has been identified as the primary mechanism governing stress relaxation and reprocessability, as confirmed by comprehensive rheological analyses. Despite substantial variations in polyol structure, initial [NCO]_0_/[OH]_0_ ratios, catalyst concentration, and catalyst identity, rapid stress relaxation—occurring within tens to hundreds of seconds—was consistently observed at temperatures ≥120 °C. Dynamic mechanical analysis (DMA) revealed a pronounced decrease in the storage modulus across the rubbery plateau, accompanied by a rubber-to-liquid transition. This behavior contrasts sharply with the Arrhenius-type viscosity evolution characteristic of vitrimer networks and instead aligns more closely with a Williams–Landel–Ferry (WLF)-type viscoelastic response. Taken together with evidence from small-molecule model reactions, high-temperature solubility experiments, and DMA measurements conducted up to 275 °C, these observations strongly support the conclusion that network relaxation in such systems is dominated by dissociative transcarbamoylation, involving reversible urethane bond cleavage and recombination [177,178,179,180,181]. Nevertheless, recent studies have demonstrated that dynamic covalent bond exchange mechanisms can be rationally engineered to balance mechanical robustness with network rearrangement, thereby enabling stress relaxation and reprocessability without compromising structural integrity [182]. From a macromolecular design perspective, precise control over polymer architecture, crosslink density, and bond lability plays a critical role in governing stress relaxation behavior, reprocessability, and long-term mechanical stability in dynamic polyurethane networks [183].

Catalyst selection plays a decisive role in the design of dissociative PU-CAN systems. Among the catalysts investigated to date, dibutyltin dilaurate (DBTDL) has long served as the benchmark in PU-CAN research due to its exceptionally high activity, even at very low loadings. Dichtel and co-workers have reported that DBTDL-catalyzed PU-CANs exhibit rapid stress relaxation in less than 40 s, with comparable activation energies and relaxation times observed across both polyether- and polyester-based urethane networks. This consistency strongly suggests that the relaxation behavior originates from dissociative urethane exchange rather than transesterification processes. In response to growing regulatory concerns regarding organotin compounds, tin-free Lewis acid catalysts such as Fe(acac)_3_ and Bi(neo)_3_ have been proposed as viable alternatives. Under optimized conditions, these catalysts have demonstrated stress-relaxation kinetics comparable to those achieved with DBTDL, underscoring their potential as environmentally benign substitutes [181,184,185]. Structure–reactivity modulation strategies based on the dissociative mechanism have also been reported by Zu and co-workers. In particular, O-phenyl carbamate linkages dissociate far more readily than their O-alkyl counterparts, and their dissociation onset temperature can be finely tuned within a 30–80 °C range through electronic substitution on the phenyl ring. In such systems, stress relaxation has been observed even at relatively low temperatures (~40 °C), while mechanical properties can be repeatedly recovered within minutes to hours at temperatures between 80 and 130 °C. The activation energy for viscous flow has been reported to range from 81 to 115 kJ·mol^−1^, and a pronounced increase in relaxation rate with increasing DABCO concentration clearly reveals dissociative reaction-controlled kinetics. These findings demonstrate that dissociation temperature, exchange rate, and effective crosslink density can be structurally engineered to precisely tailor the macroscopic behavior of PU-CANs [186,187,188]. Notably, dissociative exchange reactions are not exclusively thermally activated. In iron oxide–PU nanocomposites, localized heating induced by an external magnetic field selectively activated DBTDL-catalyzed transcarbamoylation at interfacial regions [189]. Likewise, in carbon nanotube–PU composites, microwave irradiation triggered transcarbamoylation within seconds, enabling rapid damage repair [190]. Collectively, these examples illustrate that externally stimulated, localized activation of dissociative exchange provides a powerful strategy to further expand the functional application space of PU-CANs.

To date, most reported studies on PU-based CANs have interpreted network rearrangement and stress relaxation primarily through dissociative urethane exchange mechanisms. However, several mechanistic and structural questions remain unresolved. Because many prior investigations inferred dissociative behavior primarily from normalized stress-relaxation experiments or high-temperature DMA analyses, it remains unclear which specific bonds dissociate within the network, at what stage, and to what extent. In particular, the respective contributions of urethane, urea, and biuret linkages to bond dissociation and network relaxation have not been clearly delineated. Moreover, high-temperature dissociation processes may induce secondary phenomena such as urea formation, the generation of exchange-inactive bonds, and the evolution of PU-specific soft/hard segment phase separation, as well as heterogeneous catalyst distribution. These factors can significantly influence stress relaxation behavior, processability, and long-term network stability, yet they have largely remained unaddressed in most PU-CAN studies [19]. As emphasized by Montarnal and co-workers, accurate interpretation of the rheological behavior of dissociative networks requires non-normalized stress-relaxation measurements, small-amplitude oscillatory shear experiments, and solid-state spectroscopic analyses, such as FT-IR and Raman spectroscopy. Nevertheless, experimental datasets satisfying these criteria remain scarce in the PU-CAN literature [191]. Consequently, although a qualitative consensus has emerged that dissociative urethane exchange is the dominant relaxation mechanism, quantitative validation at the network level—particularly regarding spatial homogeneity, dissociation kinetics, bond population ratios, and structural evolution during reprocessing—remains limited.

#### 3.2.2. Dissociative Urethane Exchange Enabling Foam-to-Foam Closed-Loop Recycling of PU Networks

Dissociative transcarbamoylation has attracted significant attention as a mechanism that markedly enhances the reprocessability of PU-based CANs, owing to its low activation barrier and rapid network relaxation. However, most prior studies have interpreted dissociative behavior indirectly, relying primarily on macroscopic observables such as stress relaxation times, viscosity changes, or processability. Direct experimental elucidation of dissociation equilibria and kinetics at the covalent bond level has remained limited, and systematic validation of bond-specific dissociation tendencies and network-wide homogeneity in PU systems containing mixed urethane, urea, and biuret linkages has been largely absent.

In this context, Liu and co-workers reported a set of definitive results that elucidate the intrinsic nature of dissociative mechanisms using flexible PUFs as model systems. Through model compound–based ^1^H NMR analyses, they demonstrated that, in the presence of acetoxime, urea, urethane, and biuret linkages are all converted into oxime–urethane species along with corresponding XH fragments (amines, alcohols, or ureas). By employing aromatic protons as internal standards, they conducted quantitative analysis by monitoring the time-dependent increase in the methyl signal corresponding to the oxime–urethane species, enabling direct comparison of dissociation behavior among individual covalent linkages. This analysis revealed that dissociation rates are governed by the chemical identity of the covalent bonds, with the overall extent of bond cleavage increasing linearly with time. These findings constitute the first precise experimental quantification of dissociative equilibrium kinetics in PU networks, establishing the relative dissociation constants as *K*_d_(urea) > *K*_d_(urethane) ≈ *K*_d_(biuret). Furthermore, the authors demonstrated that PUFs undergo complete swelling within only 5 s at 130 °C, providing compelling evidence that dissociative reactions proceed uniformly throughout the network without macroscopic heterogeneity. This observation indicates that rapid solvent penetration and homogeneous equilibrium establishment occur across the network prior to the onset of reaction-rate limitations, and that dissociative exchange is not constrained by phase separation or catalyst localization effects [20].

Taken together, these findings experimentally resolve many long-standing concerns surrounding dissociative exchange reactions in PU-CAN systems, including the risks of urea accumulation, irreversible side reactions, and progressive network degradation. Specifically, they provide direct answers to central questions governing dissociative PU-CAN behavior: which covalent linkages dissociate and to what extent, whether network dissociation proceeds spatially uniformly, and whether dissociation-based recycling enables structural and mechanical recovery. In doing so, this work not only delineates the fundamental limitations of dissociative mechanisms but also demonstrates a rare example of truly closed-loop polymer recycling achieved without additional catalysts, solvents, or comonomers. As such, it establishes a new benchmark for the rational design of dissociative PU-CAN systems [20]. By contrast, associative transcarbamoylation–based PHU vitrimers offer a structurally preservative platform well suited for reprocessing and foam-to-foam circular recycling, while dissociative TC–based PU-CANs are advantageous for achieving rapid stress relaxation and enhanced processability at comparatively lower temperatures. Accordingly, the design of recycling strategies for thermoset PUs should be guided by the targeted balance between thermal–mechanical stability and low-temperature reprocessability. Selective integration of associative or dissociative mechanisms, in combination with appropriate catalyst systems, thus represents a critical materials design lever for advancing closed-loop PU recycling technologies.

**Figure 18:**
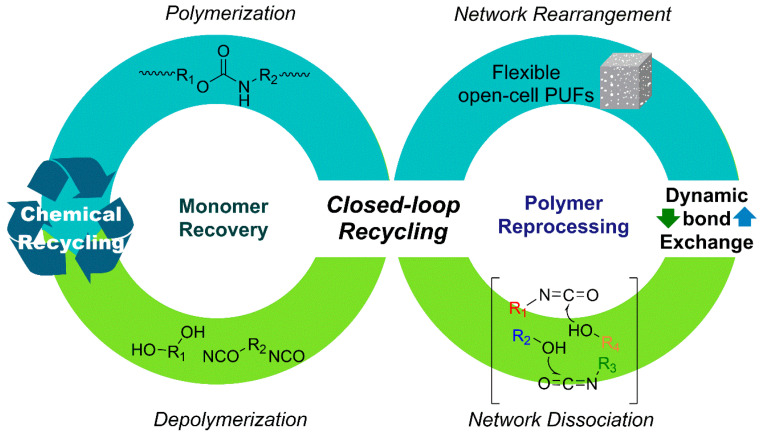
Schematic illustration of closed-loop recycling strategies for PU materials, encompassing chemical recycling through depolymerization and monomer recovery, as well as polymer reprocessing via dynamic bond exchange and network rearrangement.

This molecular- and network-level understanding of dissociative transcarbamoylation provides a critical foundation for evaluating the feasibility of closed-loop recycling in practical thermoset PUF systems (Figure 18). PUFs are biphasic porous materials composed of a continuous PU matrix and a discontinuous gaseous phase [192]. Owing to their cellular architecture and porosity, PUFs exhibit physical and mechanical behaviors that are fundamentally distinct from those of bulk thermoset resins. Foam formation is a highly dynamic, multistep process involving bubble nucleation, growth, coalescence, and eventual cell wall rupture, during which the microstructure evolves continuously [193]. In the early stages, spherical gas bubbles are dispersed within the liquid polymer matrix; as bubble density increases, polyhedral cell geometries emerge, and subsequent cell wall rupture leads to the formation of open-cell structures. The ratio of open- to closed-cell morphology plays a decisive role in determining the mechanical performance and long-term stability of low-density flexible PUFs. Based on cell size, PUFs can be classified as macrocellular (>100 μm), microcellular (1–100 μm), ultramicrocellular (0.1–1 μm), or nanocellular (0.1–100 nm). These classifications are governed by factors such as crosslink density, blowing agent type and loading, and the presence of fillers [194,195,196]. In particular, for CO_2_-blown PUFs, insufficient cell opening at the end of the expansion stage can lead to rapid gas diffusion and subsequent foam collapse, making controlled open-cell formation a key processing parameter for flexible PUFs [197,198,199]. As a result of these structural characteristics, flexible PUFs dominated by open-cell morphologies exhibit high pore connectivity and low density, providing a material environment in which thermal and external stimuli can readily propagate throughout the network. This structural openness suggests that bond dissociation and reformation at the polymer network level are less likely to remain spatially localized and can instead occur uniformly across the bulk material. Accordingly, dynamic bond-exchange mechanisms, such as dissociative transcarbamoylation, may be more effectively coupled to macroscopic property recovery in open-cell flexible PUF systems than in dense bulk thermoset PUs. From this perspective, flexible PUFs provide a particularly favorable platform for directly translating dissociative urethane exchange into large-scale reprocessability and closed-loop, foam-to-foam recycling.

**Figure 19:**
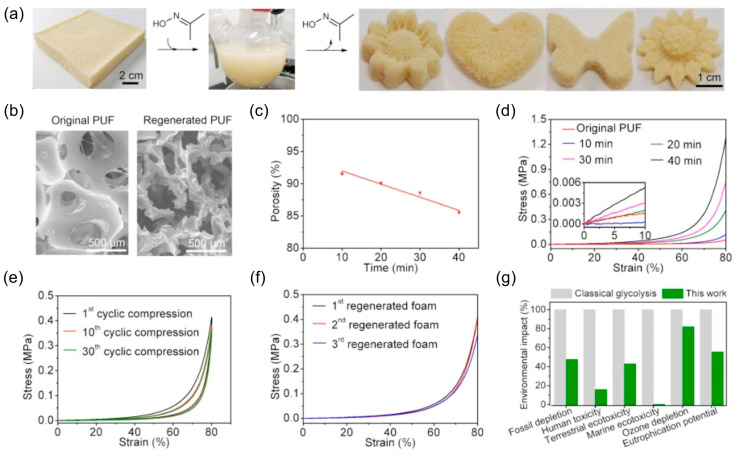
Closed-loop foam-to-foam recycling of flexible PUF via dissociative transcarbamoylation. (**a**) Photographs illustrating macroscopic reshaping and refoaming of regenerated PUF without the addition of monomers or blowing agents; (**b**) SEM images of original and regenerated PUF, showing retention of open-cell morphology; (**c**) porosity evolution of regenerated foams as a function of deconstruction time; (**d**) compressive stress–strain profiles of original and regenerated PUF prepared at varying deconstruction times; (**e**) cyclic compression behavior of regenerated PUF, demonstrating mechanical stability under repeated loading; (**f**) compressive stress–strain curves over multiple regeneration cycles, confirming reproducible mechanical performance; and (**g**) life-cycle assessment comparing the environmental impact of the dissociative recycling process with conventional glycolysis. Reprinted from [20] CC-BY-NC-ND 4.0 License.

This structural suitability was further substantiated by direct experimental evidence. Liu and co-workers demonstrated that flexible PUFs could be reshaped into various macroscopic forms via dissociative transcarbamoylation induced by acetoxime, followed by mediator removal under vacuum conditions (Figure 19a) [20]. Remarkably, both the macroscopic foam geometry and porous architecture were restored without the use of external blowing agents or additional monomers, indicating that acetoxime evaporation simultaneously facilitated pore formation and network reconstitution. SEM analysis revealed that the regenerated PUF retained a largely open-cell morphology, forming a continuous porous network in which interparticle boundaries were no longer discernible (Figure 19b). With increasing regeneration time, overall porosity gradually decreased from approximately 92% to 86% (Figure 19c), attributed to reduced particle size and increased packing density as depolymerization progressed. Notably, this evolution in porosity did not result from simple physical compression but was intrinsically linked to covalent bond reformation at the network level. Mechanical characterization further confirmed the reversibility and structural integrity of the dissociative exchange mechanism. The regenerated PUFs exhibited compressive stress–strain responses comparable to those of the original foam, with pronounced strain-hardening observed beyond 40–60% compressive strain (Figure 19d). Foams regenerated after 20 min of depolymerization displayed a low-strain elastic modulus (≈20 kPa) nearly identical to that of the pristine PUF, demonstrating effective recovery of mechanical compliance. Cyclic compression tests showed that the regenerated foams maintained stable stress–strain profiles over more than 30 loading–unloading cycles, indicating high resilience and elastic recovery (Figure 19e). Furthermore, the absence of mechanical performance degradation after up to three consecutive regeneration cycles under identical conditions provides compelling evidence that dissociative transcarbamoylation can operate repeatedly without inducing network degradation (Figure 19f). This recycling strategy also offers clear environmental advantages. Life cycle assessment indicated that foam-to-foam recycling based on dissociative exchange significantly reduces energy consumption and environmental impact compared to conventional glycolysis-based recycling processes (Figure 19g). This improvement arises from the fact that bond dissociation–recombination proceeds without the need for additional chemical reagents and enables simultaneous recovery of both the molecular network and the porous foam structure.

Collectively, the work of Liu and co-workers demonstrates that dissociative transcarbamoylation functions as more than a stress-relaxation mechanism and can be effectively translated into a practical, closed-loop foam-to-foam recycling pathway for flexible PUFs with open-cell architectures. By enabling the simultaneous restoration of the polymer network, porous morphology, and mechanical performance, this approach represents a fundamental departure from conventional depolymerization–resynthesis paradigms that currently dominate PU recycling. Instead, it establishes a new sustainability strategy based on reversible covalent exchange that preserves and reconfigures the polymer network itself, marking a decisive step toward the industrial realization of dissociative PU-CAN–based thermoset foam recycling.

## 4. Summary and Outlook

This review provides a comprehensive life-cycle perspective on sustainable PU systems, encompassing material design at the synthesis stage through to end-of-life recycling strategies. Drawing from the structural characteristics and reaction mechanisms of conventional petroleum-based PUs, recent advances in sustainable synthesis routes—including bio-based building blocks and NIPUs—are critically examined, with particular emphasis on how molecular structure design governs PU properties and processability.

For end-of-life management, two complementary recycling paradigms are highlighted. Chemical recycling approaches, such as acidolysis, aminolysis, and glycolysis, enable the selective cleavage of urethane linkages to recover polyols and amines (or isocyanate precursors), offering a closed-loop pathway at the molecular level. However, these methods face challenges related to selectivity, purification complexity, and process scalability. In parallel, dynamic covalent PU networks (PU-CANs) based on transcarbamoylation have emerged as an alternative circular strategy that retains the covalent network while enabling reprocessability. These systems extend beyond stress relaxation and self-healing to support structure-preserving recycling, including foam-to-foam closed-loop recovery of thermoset PU materials.

The diverse recycling strategies discussed above highlight both the potential and the current limitations of sustainable polyurethane technologies, which are systematically compared in Table 1. To provide a consolidated perspective on the various technological routes reviewed in this work, Table 1 summarizes and compares bio-based feedstocks, NIPU pathways, and recycling strategies in terms of technical maturity, cost implications, and environmental impact. This comparative overview highlights key trade-offs and remaining challenges toward the large-scale implementation of sustainable polyurethane systems.

Looking ahead, achieving truly sustainable PU circularity will require an integrated strategy rather than reliance on a single recycling technology. Depolymerization-based feedstock recovery and network-preserving reprocessing should be selectively combined based on material type, application requirements, and recycling objectives. Key challenges moving forward include developing a quantitative understanding of reaction mechanisms, ensuring long-term property retention over multiple recycling cycles, and rigorously evaluating process scalability using real industrial PU waste streams. By integrating synthesis, recycling mechanisms, and material performance into a unified framework, this review seeks to advance PU sustainability from a collection of isolated approaches toward a viable, circular design platform.

## Figures and Tables

**Table 1:** Comparative overview of major technological routes for sustainable polyurethane systems, evaluated in terms of technical maturity, cost implications, and carbon footprint based on literature reports.

Technological Route	Representative Examples	Technical Maturity	Cost Implication	Carbon Footprint	Key Remarks	References
Bio-based polyols (vegetable oils, lignin, sugars)	Soybean oil, castor oil, lignin-based polyols	Medium–High	Medium	Low–Medium	Sustainability strongly depends on feedstock and land use	[5,11,27,48]
NIPU synthesis (cyclic carbonate –amine)	PHUs, hybrid NIPUs, WNIPUs	Low–Medium	High	Medium	Isocyanate-free advantage, but limited kinetics and scalability	[120,129,139]
Chemical recycling (glycolysis, aminolysis)	Recycled polyols for foams and elastomers	Medium	Medium	Low	Enables closed-loop material circulation	[9,10,140]
Network- preserving recycling (PU-CAN, vitrimer-like systems)	Transcarbamoylation -based PU-CANs	Low–Medium	Medium–High	Low	Promising reprocessability, limited industrial validation	[19,20,174,176]

## Data Availability

No new data were created or analyzed in this study.
